# Chitinase 3-like protein 1 deficiency ameliorates drug-induced acute liver injury by inhibition of neutrophil recruitment through lipocalin-2

**DOI:** 10.3389/fphar.2025.1548832

**Published:** 2025-03-24

**Authors:** Ji Hye Kim, In Jun Yeo, Dong Ju Son, Sang Bae Han, Do Young Yoon, Dong Hun Lee, Jin Tae Hong

**Affiliations:** ^1^ Department of Biological Sciences, Research Center of Ecomimetics, Chonnam National University, Gwangju, Republic of Korea; ^2^ College of Pharmacy and Medical Research Center, Chungbuk National University, Cheongju, Chungbuk, Republic of Korea; ^3^ College of Pharmacy, Kyungpook National University, Daegu, Republic of Korea; ^4^ Department of Bioscience and Biotechnology, Konkuk University, Seoul, Republic of Korea

**Keywords:** CHI3L1, acetaminophen, LCN2, leukocyte recruitment, oxidative stress

## Abstract

Chitinase-3-like protein 1 (Chi3l1) is a member of the mammalian Chitinase-like protein family, and several studies reported that Chi3l1 is associated with various inflammatory diseases as well as liver diseases. Acetaminophen (APAP) is usually used for antipyretic drug, but its overdose induces acute liver injury (ALI). Several studies reported that subsequent inflammatory responses of the immune system play a critical role in the severity and outcome of APAP-induced ALI. In the present study, we investigated the role of Chi3l1 and its mechanism during APAP-induced ALI using Chi3l1 knock-out (KO) mice. We explored the function of Chi3l1 using APAP-injected KO mice and sought proteins associated with Chi3l1 through biological research data program for investigating mechanism. Liver histological analysis revealed that APAP-induced ALI was attenuated in KO mice compared to wild-type (WT) mice. We observed that APAP-induced neutrophil infiltration was decreased in the liver of KO mice compared to WT mice. To investigate this mechanism, we sought proteins potentially associated with Chi3l1 by mRNA sequencing and protein correlation analysis data. We found lipocalin-2 (Lcn2) and examined Chi3l1, Lcn2, and their relationship in the APAP-induced ALI model using recombinant proteins and antibodies. Our results suggest that Chi3l1 deficiency ameliorates APAP-induced liver injury through abrogating Lcn2-mediated neutrophil infiltration in the liver.

## 1 Introduction

Acetaminophen (APAP or paracetamol) is a frequently-used, antipyretic, and analgesic drug, but its overdose can cause severe acute liver injury (ALI) (Larsen and Wendon, 2014). The initial mechanism of ALI by APAP overdose is hepatocyte necrosis caused by increased generation of mitochondrial reactive oxygen species (ROS), mitochondrial permeabilization and dysfunction (Chambers and LoGrasso, 2011). However, several studies reported that subsequent immune responses critically play a role in the severity and outcome of APAP-induced liver injury (Antoniades et al., 2012; Krenkel et al., 2014). Several cytokines and chemokines are involved in the immune response, and various immune cells such as resident hepatic macrophages, also known as Kupffer cells (KC), infiltrating monocyte-derived macrophages, dendritic cells (DC), and lymphocytes are involved in APAP-induced liver injury (Li et al., 2022). KCs are early involved in mediating liver injury by sensing damage-associated molecular patterns (DAMPs) *via* Toll-like receptors (TLRs). This leads to the production of pro-inflammatory cytokines such as tumor necrosis factor alpha (TNF-α), interleukin 1 beta (IL-1β) and interleukin 6(IL-6) (Fisher et al., 2013), which are highly relevant for enhancing inflammation and infiltrating neutrophil and monocyte into the liver. Hepatic macrophages also attract other immune cells *via* the secretion of chemokines, such as C-C motif chemokine ligand 2(CCL2 or MCP-1) or C-X-C motif chemokine ligand 16(CXCL16) of which the first recruits monocytes into areas of necrosis (Holt et al., 2008) that, in turn, express high levels of TNF-α, IFN-γ, IL-1β, and IL-6 ^8^. The specific role of DCs in APAP-induced liver injury is still ambiguous, but a recent study reported that DCs may play a protective role in APAP-induced liver injury (Connolly et al., 2011). Lymphocytes contribute to the pathogenesis of APAP-induced liver injury by producing interferon-γ(IFN-γ) and depleting stored glutathione (GSH) in hepatocytes through the Fas/FasL system (Tinel et al., 2004), but their role in APAP-induced liver injury is still not fully elucidated.

Among the immune cells, neutrophils play an important role in ALI such as ischemia-reperfusion injury (Jaeschke et al., 1990), endotoxemia (Jaeschke et al., 1991), alcoholic hepatitis (Bautista, 2002), obstructive cholestasis (Gujral et al., 2003), alpha naphthylisothiocyanate (ANIT) toxicity (Hill et al., 1999), and APAP toxicity (Liu et al., 2006). In ALI, various inflammatory mediators such as TNF-α, IL-1β, and chemokines mediate neutrophil infiltration into the liver (Essani et al., 1995; Bajt et al., 2001), subsequently, neutrophils induce liver toxicity through increasing oxidative stress by neutrophil-derived hydrogen peroxide (Jaeschke et al., 1999), aggravated inflammatory response by neutrophilic protease (Niehorster et al., 1990), activated stress signaling pathway by neutrophil-derived IFN-γ (Wu et al., 2023), and direct hepatocellular injury by neutrophil-derived proteases (Ho et al., 1996). In APAP-induced liver injury, neutrophils infiltrate into the periphery of necrotic areas by CXCL1, CXCL2 (MIP-2), and CXCL8 secreted by KC (Zimmermann and Tacke, 2011), and IL-17A secreted by γδ T cells (Wang et al., 2013). Wang et al. reported that Interleukin 17A (IL-17A) antibody administration attenuated APAP-induced liver injury by decreasing neutrophil infiltration and interleukin 23(IL-23) levels, which is a cytokine secreted by macrophages, and required for stimulating IL-17A production from γδ T Cells (Wang et al., 2013). These results suggest that chemokines and cytokines secreted from macrophages mediate neutrophil infiltration and damage-induced liver inflammation.

Chi3l1(YKL-40) is a member of the mammalian chitinase-like protein family (Roslind and Johansen, 2009). Chi3l1 plays a role in cell proliferation, differentiation, inflammation, and immune responses (Roslind and Johansen, 2009). Recently, studies reported that Chi3l1 levels were related to liver diseases such as fibrosis, chronic hepatitis C, and chronic hepatitis B (Tao et al., 2014; Kumagai et al., 2016). In addition, Chi3l1 is closely associated with the production of chemokines and cytokines in macrophages for immune cell infiltration. Recent studies have shown that alveolar macrophages exposed to recombinant Chi3l1 produce higher levels of the proinflammatory mediators matrix metalloproteinase-9 (MMP-9), CCL2, CCL3, and CXCL2 (Letuve et al., 2008), and silencing Chi3l1 decreases secretion of pro-inflammatory molecules by macrophages (Libreros et al., 2012). Moreover, Breyne et al. reported that Chi3l1 is required for neutrophil influx against *Escherichia coli* infection in the mouse pathogenic mastitis model (Breyne et al., 2018). These results suggest that Chi3l1 may play a role in chemokine and cytokine production in macrophages necessary for neutrophil infiltration. Recently, Shan et al. reported that chi3l1 is associated with APAP-induced liver injury by promoting platelet recruitment with the liver (Shan et al., 2021). Li et al. reported that chi3l1 blocking antibody attenuated liver damage caused by APAP (Li et al., 2023). These studies suggest that Chi3l1 is important role in ALI. However, the role of Chi3l1 in the infiltration of immune cells in APAP-induced liver damage is still unknown. In this present study, we investigated the effects and possible mechanisms of Chi3l1 on APAP -induced liver injury model.

## 2 Materials and methods

### 2.1 Animals

Male and Female WT and Chi3l1 knock-out (KO) mice were obtained as described in the previous study (Im et al., 2020/01). In summary, maps depicting the wild-type Chi3l1 locus, the use of the CRISPR/Cas9 system for targeting and the predicted small deletion within exon 3 as a consequence of non-homologous end joining. WT and KO mice used had matched ages (about 3 months old). To generate APAP-induced ALI, mice were intraperitoneally (i.p.) injected with 500 mg/kg APAP, the dose used in previous stidies (Ruepp et al., 2002; Muhammad-Azam et al., 2019), and then sacrificed at 6 h. To examine the function of Chi3l1 or Lipocalin-2 (Lcn2 or NGAL) in the APAP-induced liver injury model, mice were first injected intravenously with recombinant Lcn2 (rLcn2, 10 μg/mouse, R&D, Minneapolis, MN, United States), recombinant Chi3l1 (rChi3l1, 10 μg/mouse, R&D, Minneapolis, MN, United States), with anti-Lcn2 (50 μg/mouse, Abcam, Cambridge, United Kingdom) or control antibody. After 3 h, mice were injected with APAP (300 mg/kg, i. p.), and sacrificed at 6 h. All studies received approval from and conducted in accordance with the ethical guidelines by the Chungbuk National University Animal Care Committee (CBNU-523–13–01).

### 2.2 The serum chemistry measurements

Human serum samples from 20 healthy adult donors and 20 adult patients with hepatotoxicity were obtained from Chungbuk National University Hospital and Kyung Sang University Hospital in the Republic of Korea. The characteristics of these patients are described in the supplementary (Supplementary Table S1). All studies involving human serum samples were conducted in compliance with the Declaration of Helsinki and were approved by the Ethics Committee of Chungbuk National University Medical Centre (IRB No.: CBNU-201910-BR-937–01).

Mouse serum samples were acquired through the administration of an overdose of pentobarbital (100 mg/kg) to anesthetize the mice, followed by blood collection *via* cardiac puncture. The level of aspartate transaminase (AST) and alanine transaminase (ALT) in the serum of the liver of mice were determined using an automated analyzer (7,080, Hitachi Ltd., Japan) at Laboratory Animal Research Center in Chungbuk National University.

### 2.3 Histological techniques

For histological processing, the livers were fixed in a phosphate buffer containing 10% formaldehyde and decalcified with EDTA. Fixed tissues were processed to paraffin blocks by routine methods. Specimens were sectioned at 4 μm, stained with hematoxylin and eosin (H&E) and examined for histopathological evidence of liver injury. Histopathology was scored for steatosis, inflammation, necrosis as follows: 0, normal; 1, mild changes; >2, mild to moderate severity; >3, moderate severity; 5, maximum severity.

### 2.4 Oxidative stress assay

To perform assay, liver tissues were homogenized and then normalized to protein concentration. Intracellular Hydrogen peroxides assay was performed as described in the manufacturer’s protocol (Cell biolabs, San Diego, CA). We measured the malondialdehyde (MDA) level using the TBARS assay kit (Cayman Chemical). The TBARS assay was performed as described in the manufacturer’s protocol.

### 2.5 Flow cytometry analysis

To obtain cells from the liver, the liver was washed with cold PBS until it became pale, following the cutting of the inferior vena cava was cut above the diaphragm. After removing the connective tissue and gallbladder, the liver was minced into small pieces. Subesequently, it was gently forced through a 200 mm-gauge stainless steel mesh using a sterile syringe plunger. Finally, the minced liver was suspended in 50 mL RPMI-1640 medium containing 10% FCS (pH 7.4) and GlutaMAX™-1, 25 mM HEPES. The cells from the livers of CHI3L1 WT and KO mice were screened for CD45-APC (BD Bioscience, Franklin Lakes, NJ, United States), CD11b-FITC (BD Bioscience, Franklin Lakes, NJ, United States), and Ly6G-PE (BD Bioscience, Franklin Lakes, NJ, United States).

### 2.6 Western blot analysis

Homogenized livers were lysed by protein extraction solution (PRO-PREP, iNtRONBiotechnology, Korea), which included a phosphatase inhibitor cocktail (Roche, Germany) and a protease inhibitor cocktail (Calbiochem, Germany). 30 ug of total proteins were separated by SDS-PAGE and transferred onto a PVDF membrane (Millipore, Billerica, MA). After blocking overnight with 5% skim milk, the membrane was incubated with the primary antibodies (diluted 1:1000) for 1 h at room temperature. The membranes were immunoblotted with the following primary antibodies: anti-Lcn2 (Abcam, Cambridge, MA) and anti-β-actin (Santa Cruz Biotechnology, Dallas, TX). Following the washing step with Tris-buffered saline containing 0.05% Tween-20 (TBST), the PVDF membrane was incubated with horseradish peroxidase-conjugated secondary antibodies (diluted 1:3,000) for 1 h at room temperature. Detection of Antibody binding to the blot was conducted using enhanced chemiluminescence solution (Amersham Bioscience, United Kingdom) and X-ray film (AGFA, Belgium).

### 2.7 Immunohistochemistry

All specimens were fixed in formalin and embedded in paraffin for evaluation. Subsequently, Sections of 4 μm thickness were prepared, stained with hematoxylin and eosin (H&E) and conducted by immunohistochemistry analysis using primary rabbit anti-Lcn2 (Thermo Fisher, Waltham, MA), primary rat anti-Ly6G (Abcam, Cambridge, MA), and secondary horseradish peroxidase-conjugated anti-rabbit or anti-rat antibodies.

### 2.8 Isolation of hepatocytes and kupffer cells from mouse liver

The primary mouse hepatic cells or Kupffer cells were isolated from the liver of 9-week-old, C57BL/6, male mice as described previously (Severgnini et al., 2012; Li et al., 2014). After filtering the isolated hepatocytes, they were placed into 100 mm^2^ dishes and grown in Dulbecco’s Modified Eagle Medium (DMEM) containing 10% fetal bovine serum (FBS) with 100 U/mL penicillin, and 100 mg/mL streptomycin (Gibco, Grand Island, NY, United States) at 37 °C in 5% CO_2_-humidified air. For the isolation of Kupffer cells, the filtered cells were washed Roswell Park Memorial Institute 1640 medium (RPMI 1640) and seeded into a 6-well plate at a density of 1–3 × 10^7^/well in DMEM (Hyclone, United States) with 10% FBS, 100 U/mL penicillin, and 100 mg/mL streptomycin. After then, the cells incubated for 2 h in a 5% CO_2_ atmosphere at 37 °C. After 2 h of incubation, KCs adhere to the plate and non-adherent cells can subsequently be removed by gently washing with PBS.

### 2.9 Gene-disease-mRNA-gene network analyses

The relationship between liver injury and Chi3l1 was analyzed with the ArrayExpress web server (http://www.ebi.ac.uk/arrayexpress), which provided mRNA sequencing data of human patient or mouse disease model. We selected potential genes associated with liver injury that were either up- or downregulated in the human patient or mouse liver injury model and then analyzed the relationship between those expressed genes and Chi3l1 using the GENEMANIA web server (http://genemania.org) which analyzes gene-gene networks.

### 2.10 Statistical analysis

All experiment were conducted in triplicates and replicated at least three times. Analysis of data was performed utilizing GraphPad Prism four version 4.03 software (Graph-Pad Software, La Jolla, CA). The results are presented as mean ± standard error of the mean (SEM) and assessed by one-way analysis of variance followed by the Turkey’s test. Statistical differences were considered significant at P-value <0.05.

## 3 Results

### 3.1 Chi3l1 contributes to APAP-induced liver injury

**FIGURE 1:**
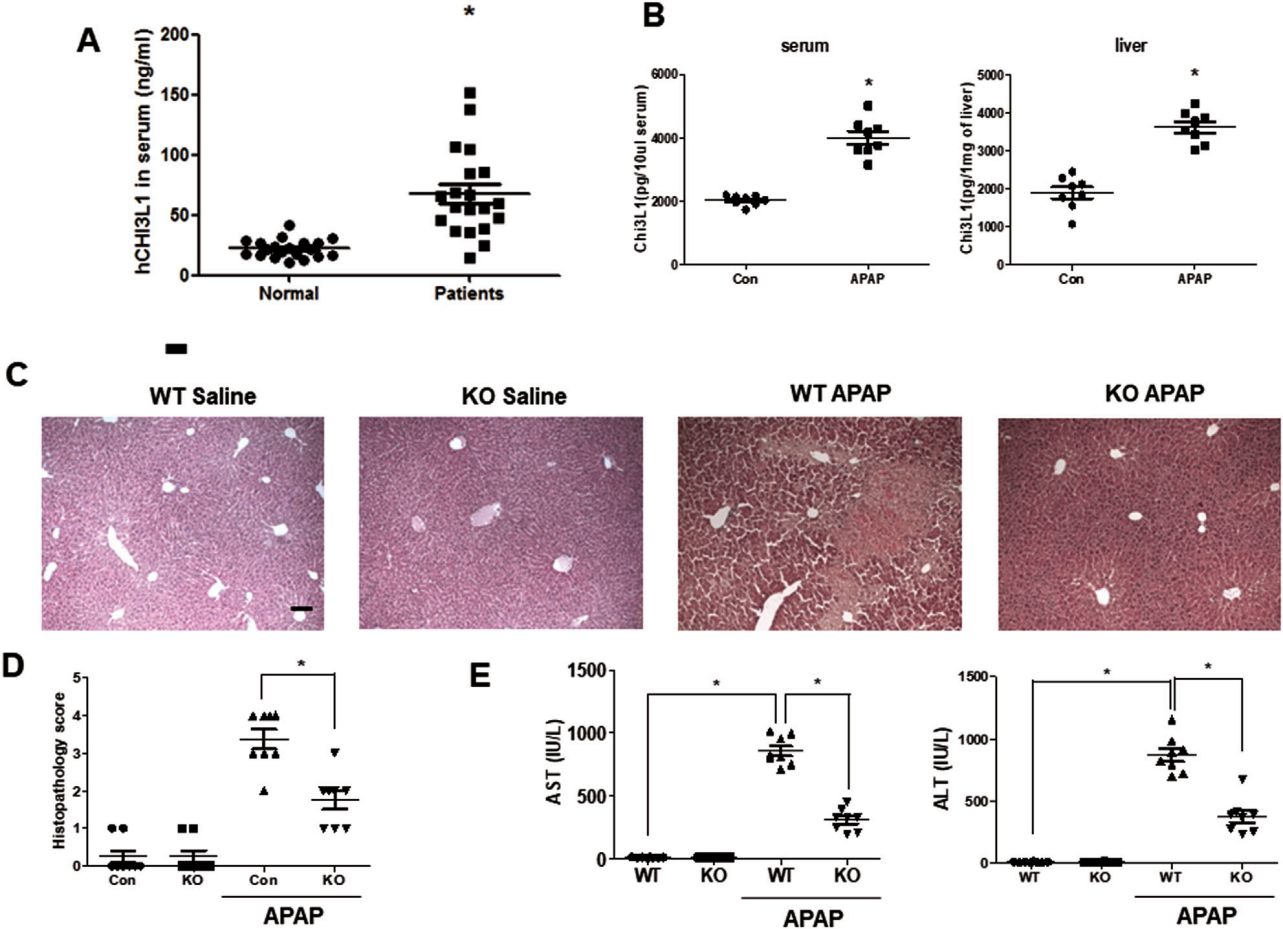
Chi3l1 deficiency shows protective effects in APAP-induced liver injury. **(A)** Serum analysis of CHI3L1 in patients with hepatotoxicity. N = 20 per group; means ± SEM, ^*^ *P* < 0.05. **(B)** The level of Chi3l1 was measured in the serum or liver of mice with or without APAP administration. N = 8 per group; means ± SEM, ^*^ *P* < 0.05. **(C)** Liver sections of WT and KO mice with or without APAP administration (500 mg/kg) were stained with hematoxylin and eosin (H&E) (Scale bars, 100 μm). **(D)** Histopathology score of liver H&E sections. **(E)** Serum AST and ALT levels in WT and KO mice with or without APAP administration (500 mg/kg). N = 8 per group; means ± SEM, ^*^ *P* < 0.05.

Several studies reported that the level of human chitinase three like protein 1(CHI3L1) increased in serum of patients with various liver diseases, including chronic liver disease and acute liver disease (Kumagai et al., 2016; Shan et al., 2021; Lee et al., 2011; Wang et al., 2020). Thus, we measured the level of serum CHI3L1 in patients with hepatotoxicity and found that it was significantly increased compared to normal donors (Figure 1A). Similarly, the levels of Chi3l1 in mouse serum and liver were dramatically increased by APAP administration (Figure 1B). Interestingly, we found that KO mice deficient in Chi3l1 were less susceptible to APAP-induced liver injury than WT mice. Histological analysis revealed massive damage in the livers of WT mice caused by APAP administration, compared to the livers of KO mice (Figures 1C,D). In accordance with the histological analysis, the AST and ALT levels induced APAP in serum were decreased in the KO mice (Figure 1E).

### 3.2 Chi3l1 is required for APAP-induced hepatic neutrophil infiltration

**FIGURE 2:**
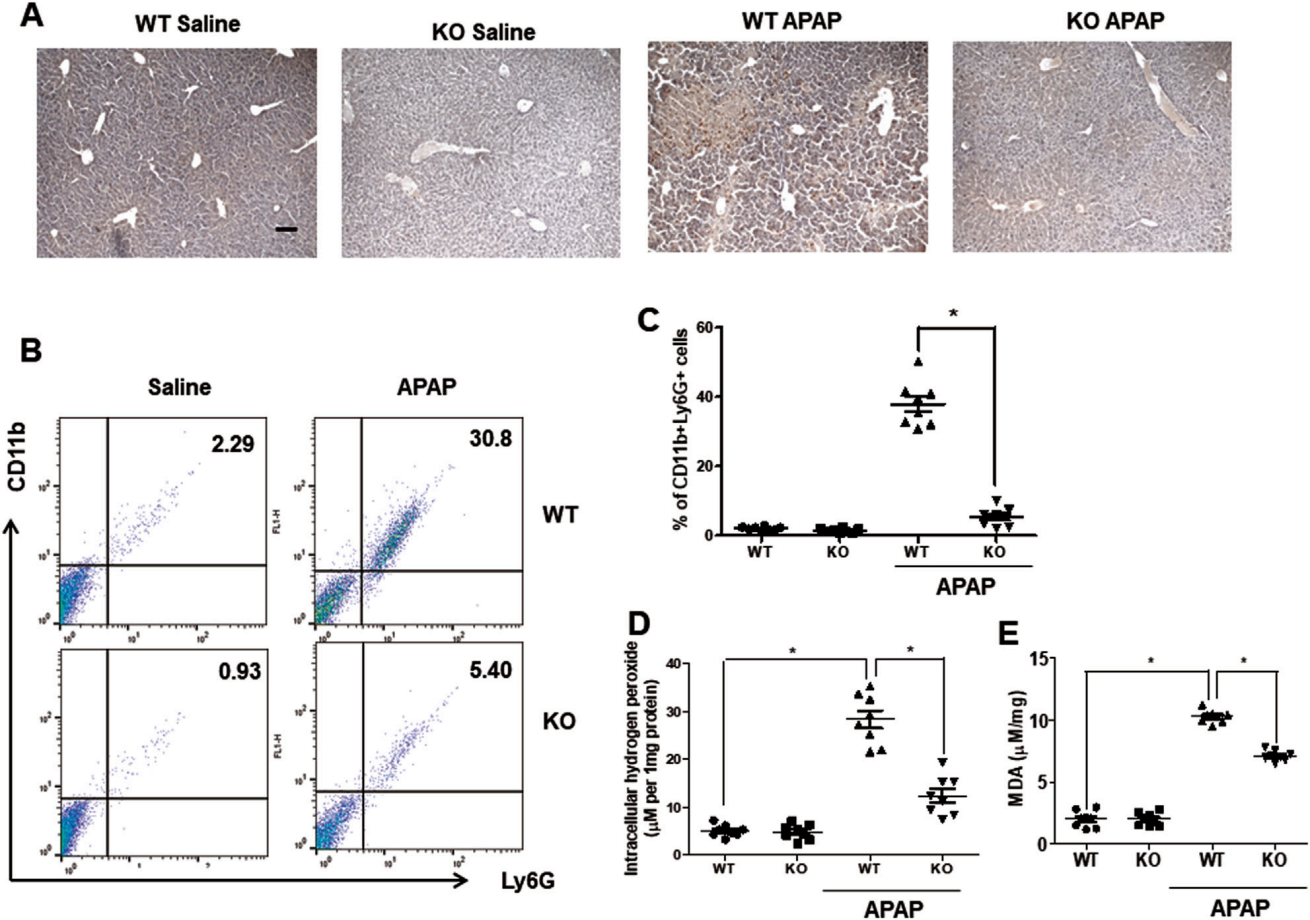
Chi3l1 deficiency abrogates recruitment of neutrophils into the liver by APAP overdose. **(A)** Immunohistochemistry of infiltrated neutrophils (Ly6G) in the livers of WT and KO mice with or without APAP administration (500 mg/kg) (Scale bars, 100 μm). Ly6G is marked with a brown dot. **(B)** Leukocytes were isolated from the livers of mice. The total CD11b+Ly6G + cells among all hepatic leukocytes were analyzed by flow cytometry. **(C)** Statistical analysis of the percentage of neutrophils in the hepatic leukocytes. N = 8 per group; means ± SEM, ^*^ *P* < 0.05. **(D)** Intracellular hydrogen peroxide levels and **(E)** MDA levels in the liver of WT and KO with or without APAP administration (500 mg/kg). N = 8 per group; means ± SEM, ^*^ *P* < 0.05.

Neutrophils are the first responders to tissue injury (Graubardt et al., 2017), and the major constituent of leukocytes infiltrating the liver after APAP administration (Wang et al., 2013). Chi3l1 is closely associated with neutrophil infiltration (Breyne et al., 2018) thus, we measured the neutrophil population in the livers of WT and KO mice after APAP administration. Immunohistochemistry data reveal that, after APAP injection, neutrophils infiltrated the hepatic injury region in the livers of WT mice, whereas infiltrated neutrophils were almost completely mitigated in the livers of KO mice (Figure 2A). FACS analysis also showed that APAP-induced infiltrating neutrophils (CD11+Ly6G+) were decreased in the livers of KO mice compared to WT mice (Figures 2B,C). Myeloperoxydase (MPO), an indicator of neutrophil infiltration, is also decreased in the livers of KO mice compared to WT mice (Supplementary Figure S1). In contrast to neutrophils, macrophage migration was not affected by *Chi3l1* deletion in the APAP-induced livers (Supplementary Figure S2). Neutrophils induce liver toxicity through oxidative stress by neutrophil-derived hydrogen peroxide, thus we investigated the oxidative stress in the livers of APAP-injected WT and KO mice. Our data reveal that the levels of hydrogen peroxide were elevated in the livers of APAP-injected WT mice whereas they were reduced in the livers of APAP-injected KO mice (Figure 2D). Accordingly, the level of MDA, a naturally occurring product of lipid peroxidation and an marker of oxidative stress, in the liver was also induced by APAP in WT mice; however, it was lower in the livers of APAP-injected KO mice (Figure 2E).

### 3.3 APAP-induced Chi3l1 expression is not hepatocytes, but in the macrophages, and its expression induces chemokines and cytokines required for neutrophil infiltration

**FIGURE 3:**
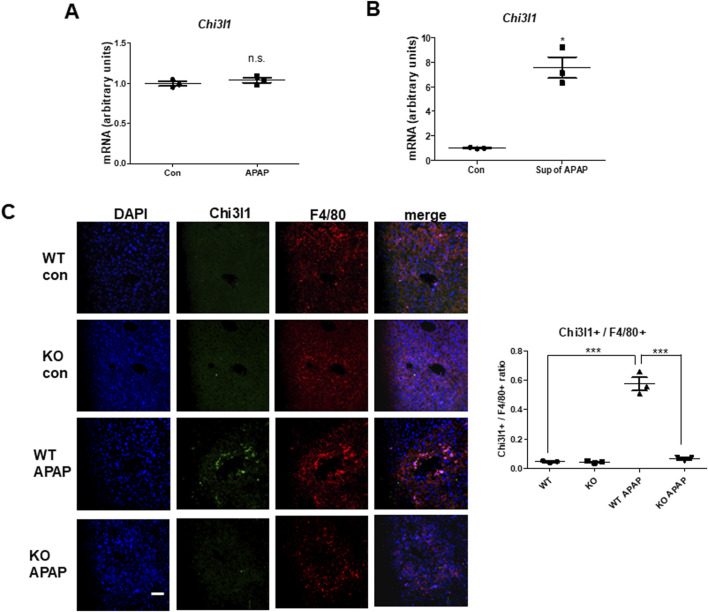
Chi3l1, which is induced by APAP administration, was expressed in macrophages, not hepatocytes. **(A)** Chi3l1 mRNA expression was measured in primary hepatocytes treated with or without APAP. **(B)** Kupffer cells were isolated from WT mice and then treated with the supernatant of APAP-treated hepatocytes (sup of APAP) or PBS-treated hepatocytes (Con). Then, Chi3l1 mRNA expression was measured. **(C)** Liver sections were triple-stained with Chi3l1 antibody (Alexa 488; green), F4/80 antibody (Alexa 568; red), and DAPI (nuclear counterstain; blue), and the images were analyzed by confocal laser-scanning microscopy (Scale bars, 50 μm) Values of chi3l1+/F4/80+ cells ratio are expressed as the means ± SEM. ^***^ *P* < 0.001.

**FIGURE 4:**
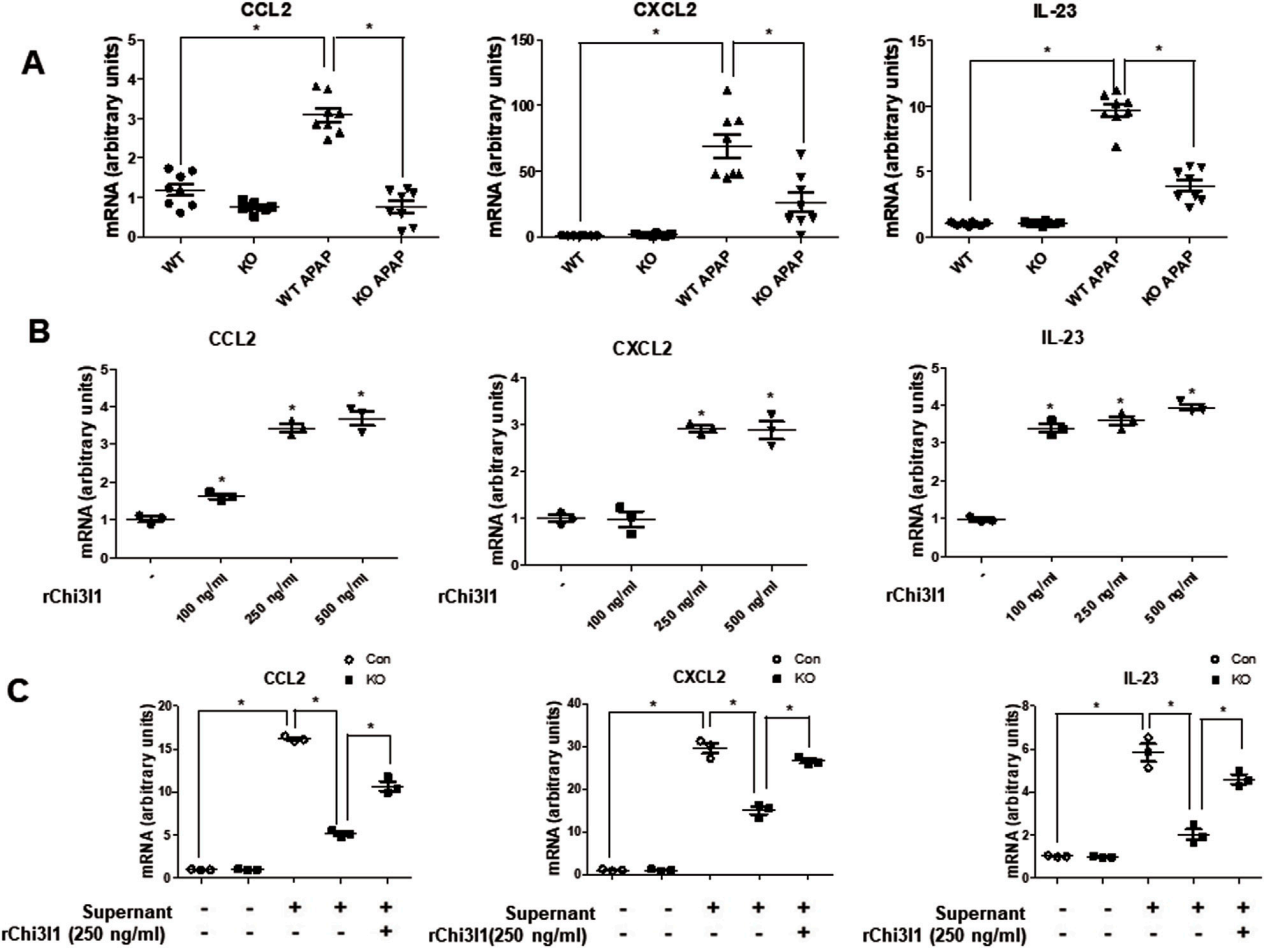
The gene expression of *Ccl2, Cxcl2,* and *Il-23* which are involved in neutrophil recruitment. **(A)** The mRNA expression of *Ccl2, Cxcl2,* and *Il-23* in the liver of WT and KO with or without APAP administration (500 mg/kg). N = 8 per group; means ± SEM, ^*^ *P* < 0.05. **(B)** The mRNA expression of *Ccl2, Cxcl2,* and *Il-23* in Kupffer cells isolated from WT mice liver and dose-dependently treated with rChi3l1. Values are expressed as the mean ± SEM of three different experiments conducted in triplicates. ^*^ *P* < 0.05. **(C)** mRNA expression of *Ccl2, Cxcl2,* and *Il-23* in Kupffer cells isolated from WT or KO mice liver pretreated with rCHI3L1 and then treated with or without supernatant of APAP-treated mouse hepatocytes. Values are expressed as the mean ± SEM of three different experiments conducted in triplicates. ^*^ *P* < 0.05.

Since previous studies reported that macrophages are the major cells expressing Chi3l1 (Zhao et al., 2023), we wondered which cells in the liver have the most Chi3l1 expression and production after APAP administration. To determine whether the expression of Chi3l1 could be induced by APAP in hepatocytes, we isolated mouse primary hepatocytes from WT mouse liver and determined the amount of Chi3l1 mRNA before and after APAP treatment. The levels of Chi3l1 mRNA were not changed by APAP treatment in mouse primary hepatocytes. However, Chi3l1 mRNA levels were significantly increased in Kupffer cells from WT mouse liver after treatment with the supernatant of APAP-treated mouse hepatocytes (Figures 3A,B). In addition, immunofluorescence data showed that the major site of APAP-induced Chi3l1 expression was in macrophages, labeled with mice macrophage maker, F4/80 (Figure 3C). These results suggest that Chi3l1 expression was not induced by APAP in hepatocytes, but its expression was induced in macrophage by secreted factors such as DAMPs from APAP-damaged hepatocytes. Next, we examined chemokines and cytokines required for neutrophil infiltration by Chi3l1 in APAP-induced liver injury. We found that the levels of APAP-induced IL-17 and IL-23, which are key factors for neutrophil infiltration, were abrogated in the livers of Chi3l1 KO mice (Supplementary Figure S3). The mRNA and protein levels of CCL2, CXCL2, and IL-23 were increased by APAP injection in the livers of WT mice, but these elevated mRNA levels were reduced in APAP-induced livers of KO mice (Figure 4A; Supplementary Figures S3, S4). Since Chi3l1 is expressed in macrophages and Kupffer cells, and resident macrophages are the main cells recruiting neutrophils to the liver, we isolated Kupffer cells from liver of WT mice then investigated the role of Chi3l1 in the expression of CCL2, CXCL2, and IL-23 which are all associated with neutrophils recruitment (Wang et al., 2013; Moles et al., 2014). The mRNA level of CCL2, CXCL2, and IL-23 were further increased by dose-dependent, rChi3l1 treatment in Kupffer cells from the livers of WT mice (Figure 4B). In contrast, after treatment of Kupffer cells with the supernatant of APAP-treated mouse hepatocytes, the mRNA levels of CCL2, CXCL2, and IL-23 were reduced in Kupffer cells from the livers of KO mice compared to WT mice, but these levels were restored by rChi3l1 treatment (Figure 4C). These results suggest that Chi3l1 plays a role in chemokine and cytokine expression in macrophages that is required for neutrophil infiltration.

### 3.4 Lcn2 is associated with Chi3l1 in APAP-induced liver injury

**FIGURE 5:**
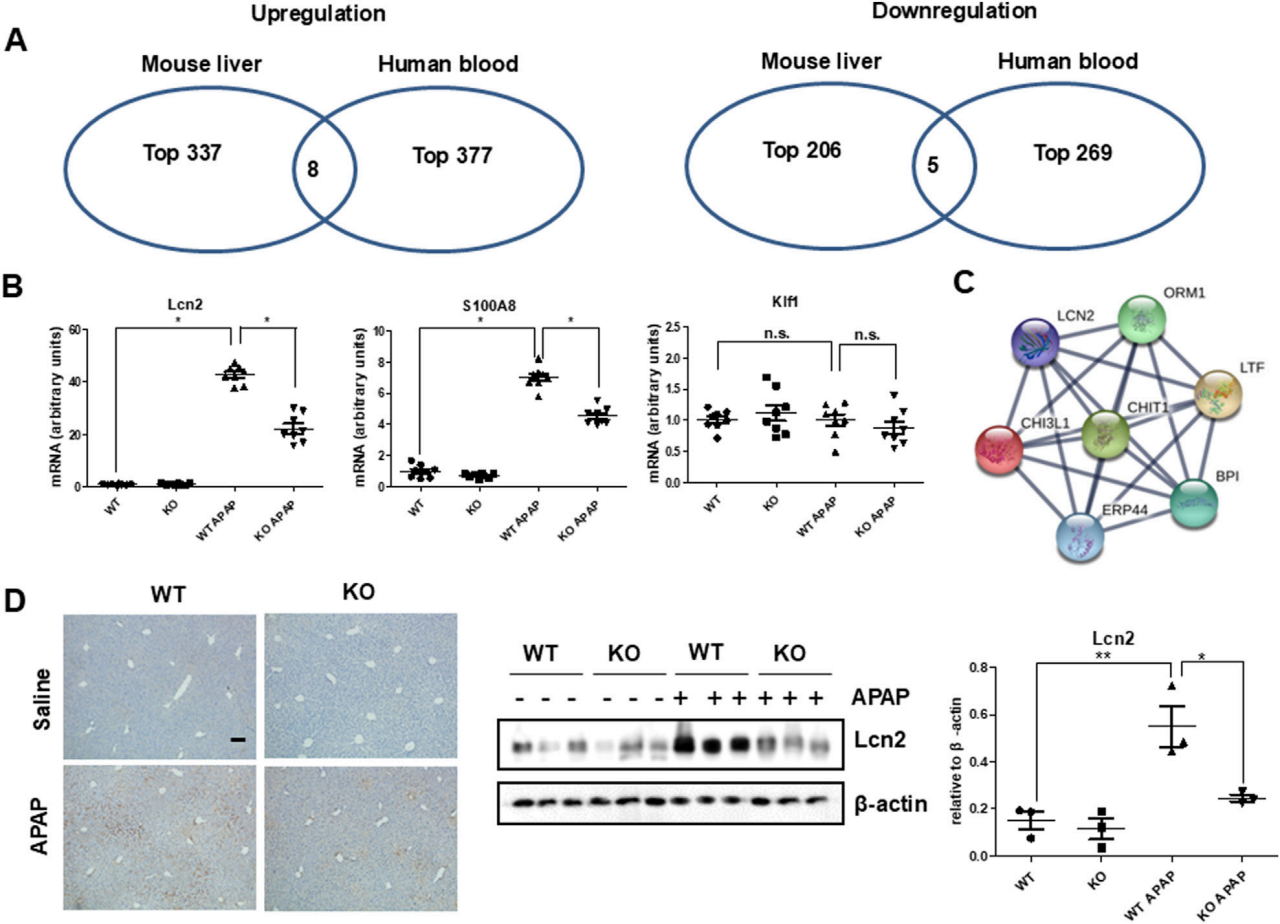
Strategy for target regulated by Chi3l1. **(A)** Select co-upregulated or co-downregulated genes from RNA sequencing analysis of mouse liver with or without APAP administration and human blood from a patient with APAP exposure or a healthy donor. **(B)** The mRNA expression of Lcn2, S100A8, and Klf1 in the liver of WT and KO with or without APAP administration (500 mg/kg). N = 8 per group; means ± SEM, ^*^ *P* < 0.05. **(C)** STRING network analysis with Chi3l1 and Lcn2. **(D)** Immunohistochemistry and immunoblot of Lcn2 in the liver of WT and KO mice with or without APAP administration (500 mg/kg) (Scale bars, 100 μm). Values are expressed as the means ± SEM. ^*^ *P* < 0.05, ^**^ *P* < 0.01.

Next, we investigated target proteins regulated by Chi3l1 in APAP-induced liver injury. Firstly, we analyzed RNA sequencing data, which are either data from the livers of mice orally treated with APAP (Accession No. E-GEOD-51969) or human blood samples of patients treated with APAP (Accession No. E-GEOD-70786), from ArrayExpress (www.ebi.ac.uk/arrayexpress) (Figure 5A). The analysis revealed that eight genes were upregulated and five genes were downregulated in both APAP-treated mouse liver and human blood samples. Then, we analyzed whether these genes are associated with Chi3l1 using GENEMANIA. We found that three upregulated genes (Lcn2, S100A8, Klf1) were correlated with Chi3l1 (Supplementary Figure S5). Thus, we validated the expression of these three genes using quantitative PCR in APAP-induced livers of WT or KO mice. The elevated mRNA expression of *Lcn2* and *S100A8* by APAP administration were abrogated in the livers of Chi3l1 KO mice, but the mRNA expression of Klf1 exhibited no changes due to APAP administration and loss of Chi3l1 (Figure 5B). We focused on the Lcn2 because STRING network analysis revealed a significant correlation between Lcn2 and Chi3l1 (Figure 5C). To validate if the expression of Lcn2 is affected by Chi3l1, we investigated the Lcn2 protein expressed in macrophages treated with rChi3l1. We show that Lcn2 expression was increased by dose-dependent, rChi3l1 treatment (Supplementary Figure S6). Moreover, expression of Lcn2 protein was significantly decreased in the APAP-treated livers of Chi3l1 KO mice compared to WT mice (Figure 5D). These data suggest that Lcn2 might be induced by Chi3l1.

### 3.5 Lcn2 aggravates APAP-induced liver injury in Chi3l1 KO mice

**FIGURE 6:**
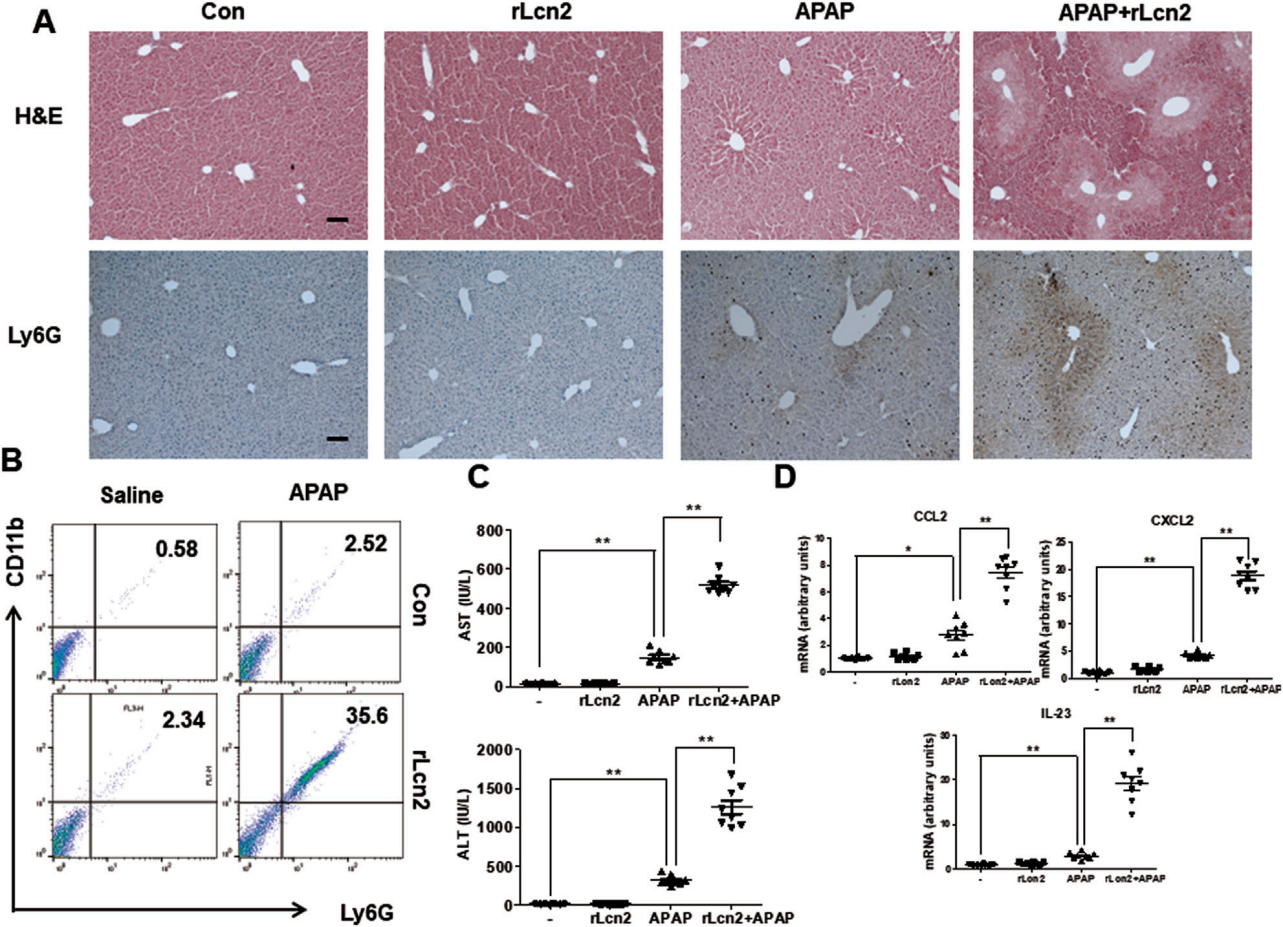
Lcn2 showed enhanced effects on APAP-induced liver injury in mice. **(A)** Hematoxylin and eosin (H&E) staining and immunohistochemistry of Ly6G of liver sections from KO mice injected with or without rLcn2 (10 μg/mouse) under APAP administration (300 mg/kg) (Scale bars, 100 μm). **(B)** Leukocytes were isolated from the liver. The total CD11b+Ly6G + cells among all hepatic leukocytes were analyzed by flow cytometry. **(C)** Serum AST and ALT levels in mice injected with rLcn2 (10 μg/mouse) in APAP administration (300 mg/kg) or not. N = 8 per group; means ± SEM, ^*^ *P* < 0.05. **(D)** The mRNA expression of *Ccl2, Cxcl2,* and *Il-23* in the liver of KO mice injected with rLcn2 (10 μg/mouse) in APAP administration (300 mg/kg) or not. N = 8 per group; means ± SEM, ^*^ *P* < 0.05, ^**^ *P* < 0.01.

Because APAP-induced Lcn2 expression was inhibited in Chi3l1 KO mice, we administered exogenous rLcn2 into Chi3l1 KO mice injected with or without APAP to investigate the effect of Lcn2 in APAP-treated Chi3l1 KO mice. The rLcn2 administration did not induce liver injury, but it weakly recruited neutrophils to the liver (Figure 6). However, attenuated APAP-induced liver injury in the Chi3l1 KO mice was aggravated by rLcn2 injection as well as increased neutrophil infiltration to the liver (Figures 6A,B). In accordance with the histological analysis, the APAP-induced CCL2, CXCL2, and IL-23 in the liver and AST, ALT serum levels were increased by rLcn2 in KO mice (Figures 6C,D). These results suggest that inhibited APAP-induced liver injury and neutrophil infiltration in Chi3l1 KO mice is mediated by Lcn2.

**FIGURE 7:**
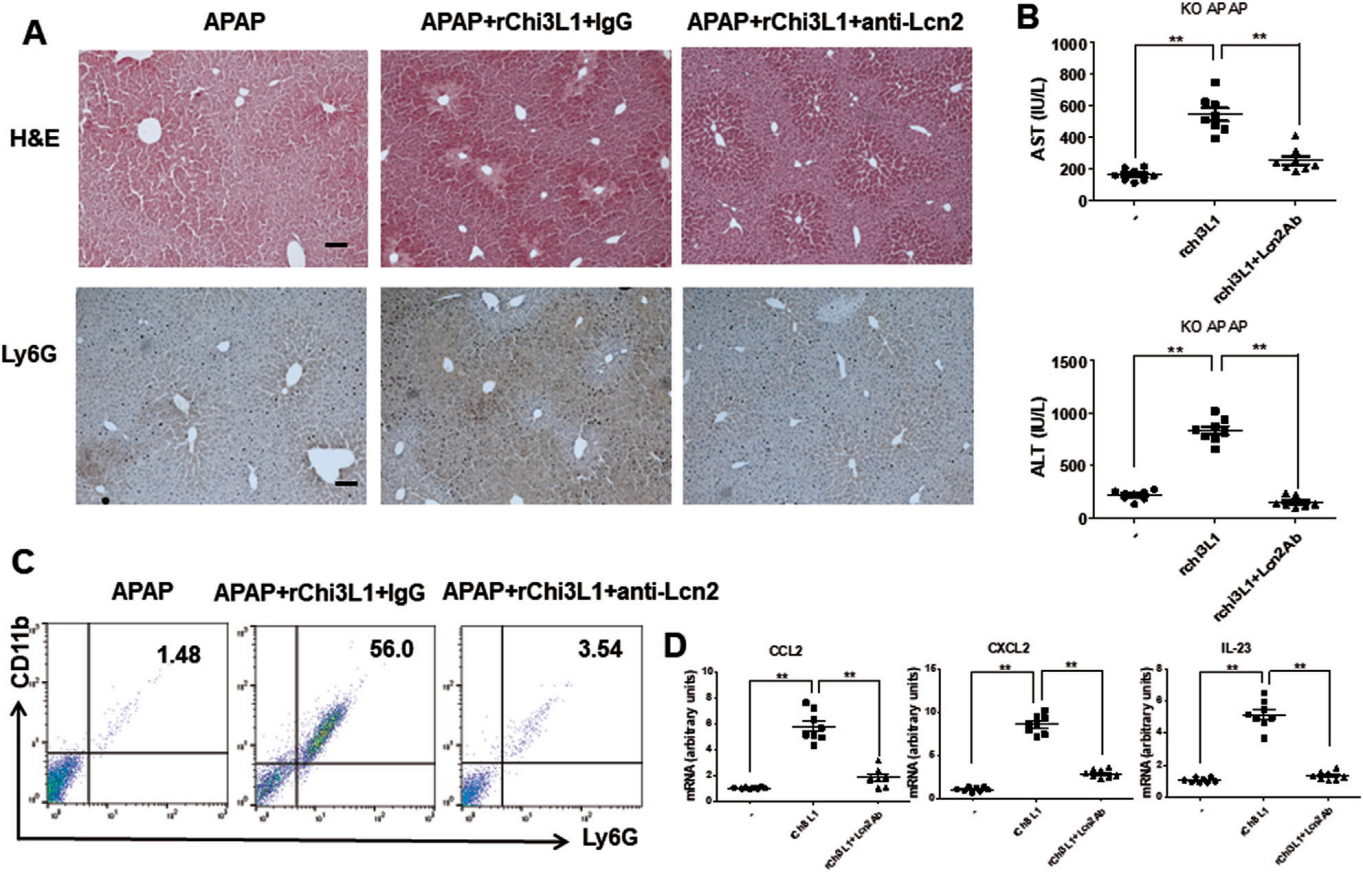
Lcn2 antibody recovered in exacerbated liver injury by rChi3l1 on APAP administration. **(A)** Hematoxylin and eosin (H&E) staining and immunohistochemistry of Ly6G of liver sections in rLcn2-and APAP-administrated KO mice pre-injected with anti-Lcn2 (50 μg/mouse) or anti-IgG (300 mg/kg) (Scale bars, 100 μm). **(B)** Serum AST and ALT levels in rLcn2-and APAP- administrated KO mice pre-injected with anti-Lcn2 (50 μg/mouse) or anti-IgG (300 mg/kg). N = 8 per group; means ± SEM, ^**^ *P* < 0.01. **(C)** Leukocytes were isolated from the liver. The total CD11b+Ly6G + cells among all hepatic leukocytes were analyzed by flow cytometry. **(D)** The mRNA expression of *Ccl2, Cxcl2,* and *Il-23* in the liver of rLcn2-and APAP- administrated WT mice pre-injected with anti-Lcn2 (50 μg/mouse) or anti-IgG (300 mg/kg). N = 8 per group; means ± SEM, ^**^ *P* < 0.01.

To further demonstrate that Chi3l1 can exacerbate APAP-induced liver injury through Lcn2, we treated Chi3l1 KO mice with rChi3l1 while neutralizing Lcn2. The data reveal that rChi3l1-induced deterioration of APAP-induced liver injury was abrogated by Lcn2 neutralization (Figures 7A,B). Moreover, APAP-induced neutrophil infiltration as well as CCL2, CXCL2, and IL-23 expression in the liver by rChi3l1 were inhibited by Lcn2 neutralization (Figures 7C,D). These results suggest that Lcn2, induced by Chi3l1, drives neutrophil infiltration in APAP-induced liver injury.

## 4 Discussion

CLPs (Chitinase like proteins) are proteins that are structurally similar to chitinase and bind to chitin but cannot be broken down (Yu et al., 2024). Studies of CLPs have revealed the types and various functions, for example, involved in immune response control and pathogen detection (Yu et al., 2024). Among them, chi3l1 is associated with cancer as well as non-neoplastic diseases characterized by inflammation such as arthritis, infectious disease, inflammatory bowel disease, kidney injury, and liver disease (Kumagai et al., 2016; Kjaergaard et al., 2014). In cancer, Chi3l1 plays a role in metastasis and is increased in serum; however, the function of Chi3l1 in various diseases remains unclear. Recently, a study was reported that CHI3L1 is involved in platelet recruitment and damage of the liver induced by APAP (Shan et al., 2021). However, many studies are still needed on the association between CHI3L1 and acute liver damage. To investigate this, we used Chi3l1 KO mice and employed an APAP-induced liver injury model which is frequently used for drug-induced hepatotoxicity.

APAP is a commonly-used drug, but its overdose leads to hepatocyte cell death by increasing oxidative stress and mitochondrial dysfunction during metabolism of APAP. Several recent studies reported the pathogenesis of APAP-induced liver injury. They revealed that not only acute hepatocytes necrosis but also subsequent inflammatory responses can critically affect the severity of the disease (Antoniades et al., 2012). Neutrophil-mediated liver injury has been reported in experimental drug/chemical-induced animal models such as ethanol toxicity, ANIT, and APAP (Ramaiah and Jaeschke, 2007; Smith et al., 1998). In drug/chemical-induced liver injury models, inflammatory mediators stimulated by oxidative stress and tissue injury lead to neutrophil invasion that often worsens the liver damage. Although it is still controversial whether neutrophils directly aggravate the course of APAP-induced liver injury (Jaeschke, 2008), several studies indicated that neutrophils can directly mediate hepatocyte necrosis in APAP-induced liver injury by neutrophil-derived ROS (Marques et al., 2012). In addition, it has been reported that decrease in neutrophil infiltration attenuates drug-induced acute liver damage (Matsuo et al., 2023). In our study, we found that Chi3l1-deficient mice had attenuated APAP-induced liver injury and abrogated neutrophil infiltration as well as decreased liver oxidative stress. In rodent experiments, a number of neutrophils are recruited into the liver at 4–24 h after treatment with a high dose of APAP (Lawson et al., 2000), and pretreatment with a neutrophil antiserum or anti-Gr-1 monoclonal antibody significantly attenuated hepatic neutrophil accumulation and liver injury after APAP administration (Liu et al., 2006; Smith et al., 1998). APAP-induced necrotic hepatocytes release DAMPs, such as high-mobility group box 1 (HMGB1), heat shock proteins, DNA, and cyclophilin A (Martin-Murphy et al., 2010; Dear et al., 2011). These DAMPs activate KCs, which are the resident hepatic macrophages, that in turn recruit neutrophils through various inflammatory mediators or γδ T cells (Wang et al., 2013). Our data show that Chi3l1 was not induced in hepatocytes and rChi3l1 treatment did not affect APAP-induced hepatic cell death. However, cell supernatant from APAP-treated hepatocytes induced Chi3l1 expression in macrophages, and immunofluorescence data revealed that Chi3l1 was mainly expressed in macrophages in the APAP-treated liver. These results suggest that Chi3l1 is not associated with APAP-induced cell death in hepatocytes, but the source of increasing Chi3l1 by APAP is maybe associated with macrophage-mediated liver injury. Furthermore, previous studies have reported the association of Chi3l1 with M1 macrophage and M2 macrophage (Zhao et al., 2023; Higashiyama et al., 2019). M2 secretes a large amount of CHI3L1 and CHI3L1 promotes M2 polarization in many diseases (Zhao et al., 2023). On the other hand, it has also been reported that CHI3L1 deficiency inhibit apoptosis of M1, but not apoptosis of M2^53^. Accordingly, the association between CHI3L1 and macrophage still had an argument. In addition, it is known that the balance of M1/M2 is important in liver disease in many works (Wang et al., 2021). Therefore, further understanding of the association of Chi3l1 and M1/M2 is required in drug-induced acute liver disease.

Next, we investigated the function of Chi3l1 in APAP-induced liver injury. To study this, we first performed a cytokines/chemokines array and found that APAP-induced IL-17 and IL-23, which are key factors for neutrophil infiltration, were abrogated in the livers of Chi3l1-deficient mice. CHI3L1 is closely associated with the production of chemokines and cytokines in macrophages for immune cell infiltration (Letuve et al., 2008; Libreros et al., 2012; Breyne et al., 2018). Thus, we validated the levels of CCL2, CXCL2, and IL-23, which are involved in neutrophil infiltration, in the APAP-treated livers and macrophages. We found that they were not increased in the macrophages and livers of Chi3l1 KO mice. These results suggest that Chi3l1 may affect macrophage activation to produce chemokines and cytokines necessary for neutrophil infiltration.

Previous studies have shown that chi3l1 increased platelet recruitment by increasing podoplanin *via* receptor CD44 in macrophages, leading to platelet involvement in tissue damage in the early stages to AILI (Shan et al., 2021). Our data showed that neutrophil infiltration into the liver increased by chi3l1 derived from KC and was involved in ALI. These finding suggest that chi3l1 plays an important role in contributing to AILI exacerbation by inducing tissue damage through platelet and neutrophil recruitment. Platelet is involved in neutrophil recruitment to the liver (Morris and Chauhan, 2022), and platelet depletion within necrotizing foci in APAP-treated mice tends to reduce neutrophil accumulation (Miyakawa et al., 2015). Since these finding suggest that interactions between them are likely involved in promoting neutrophil accumulation in AILI, further studies are needed to reveal the mechanism by which platelet-neutrophils interactions causes APAP-induced liver damage.

Second, we investigated what protein is associated with Chi3l1 in APAP-induced liver injury. We thus downloaded RNA sequencing data of APAP-treated mice and patients from ArrayExpress site (www.ebi.ac.uk/arrayexpress). We found that *Lcn2* was among the upregulated genes in APAP-treated mice and humans and was associated with Chi3l1. We then observed that Lcn2 was increased in the livers of APAP-treated mice but not in the APAP-treated Chi3l1 KO mice. Lcn2, also known as neutrophil gelatinase associated lipocalin (NGAL), may be an early biomarker of liver inflammation because it is highly upregulated in response to inflammation, injury, and metabolic stress in the liver (Xiao et al., 2017; Wieser et al., 2016). Several studies reported that Lcn2 may act to recruit neutrophils to the site of inflammation in various tissues (Xu et al., 2015). Moreover, Lcn2 is associated with neutrophilic inflammation and neutrophil-macrophage crosstalk in liver diseases (Wieser et al., 2016; Ye et al., 2016). Thus, we examined the relationship between Lcn2 and Chi3l1 in the livers of APAP-treated mice. In the present study, we found that Lcn2 was induced by Chi3l1, and the attenuated liver injury by Chi3l1 deficiency was exacerbated by Lcn2 administration in APAP-treated mouse liver. Moreover, exacerbated liver injury and elevated neutrophil infiltration by rChi3l1 were reduced through neutralizing Lcn2 by antibody treatment in APAP-treated Chi3l1 KO mouse liver. These results suggest that Lcn2 is induced by Chi3l1 and is associated with neutrophil infiltration in APAP-induced liver injury.

In summary, our results indicate that Chi3l1 induces Lcn2, which induced neutrophil infiltration leading to liver injury by APAP, and reveal a novel mechanism in which chi3l1 is involved in neutrophil infiltration. These studies support treatment strategies targeting chi3l1 in APAP-induced liver injury and suggest that Lcn2 inhibition has a protective effect on liver damage by APAP.

## Data Availability

The original contributions presented in the study are included in the article/Supplementary Material, further inquiries can be directed to the corresponding author.

## Glossary

ALT: alanine transaminase
AST: aspartate transaminase
ALI: acute liver injury
ANIT: alpha naphthylisothiocyanate
APAP: Acetaminophen
Chi3l1: Chitinase-3-like protein 1
CHI3L1: human chitinase three like protein 1
CCL: C-C motif chemokine ligand
CXCL: C-X-C motif chemokine ligand CLPs: chitinase like proteins
DAMPs: damage-associated molecular patterns
DC: dendritic cells
DMEM: Dulbecco’s Modified Eagle Medium
FBS: fetal bovine serum
GSH: glutathione
HMGB1: high-mobility group box one
I.p.: intraperitoneally
IL: Interleukin
IFN-γ: Interferon-γ
KO: Chi3l1 knock-out
KC: Kupffer cells
Lcn2: lipocalin-2
MDA: malondialdehyde
MMP-9: matrix metalloproteinase-9
MPO: myeoloperoxydase
NGAL: neutrophil gelatinase associated lipocalin
rLcn2: recombinant lipocalin-2
rChi3l1: recombinant Chitinase-3-like protein 1
ROS: reactive oxygen species
RPMI 1640: Roswell Park Memorial Institute 1640 medium
TLRs: Toll-like receptors
TNF-α: Tumor necrosis factor alpha
TBST: Tris-buffered saline containing 0.05% Tween-20
WT: wild-type
