# Supplementary figures and images for: Chitinase 3-like protein 1 deficiency ameliorates drug-induced acute liver injury by inhibition of neutrophil recruitment through lipocalin-2
Source: Front Pharmacol. 2025 Mar 24;16:1548832. doi: 10.3389/fphar.2025.1548832 (PMC11973357; doi:10.3389/fphar.2025.1548832)

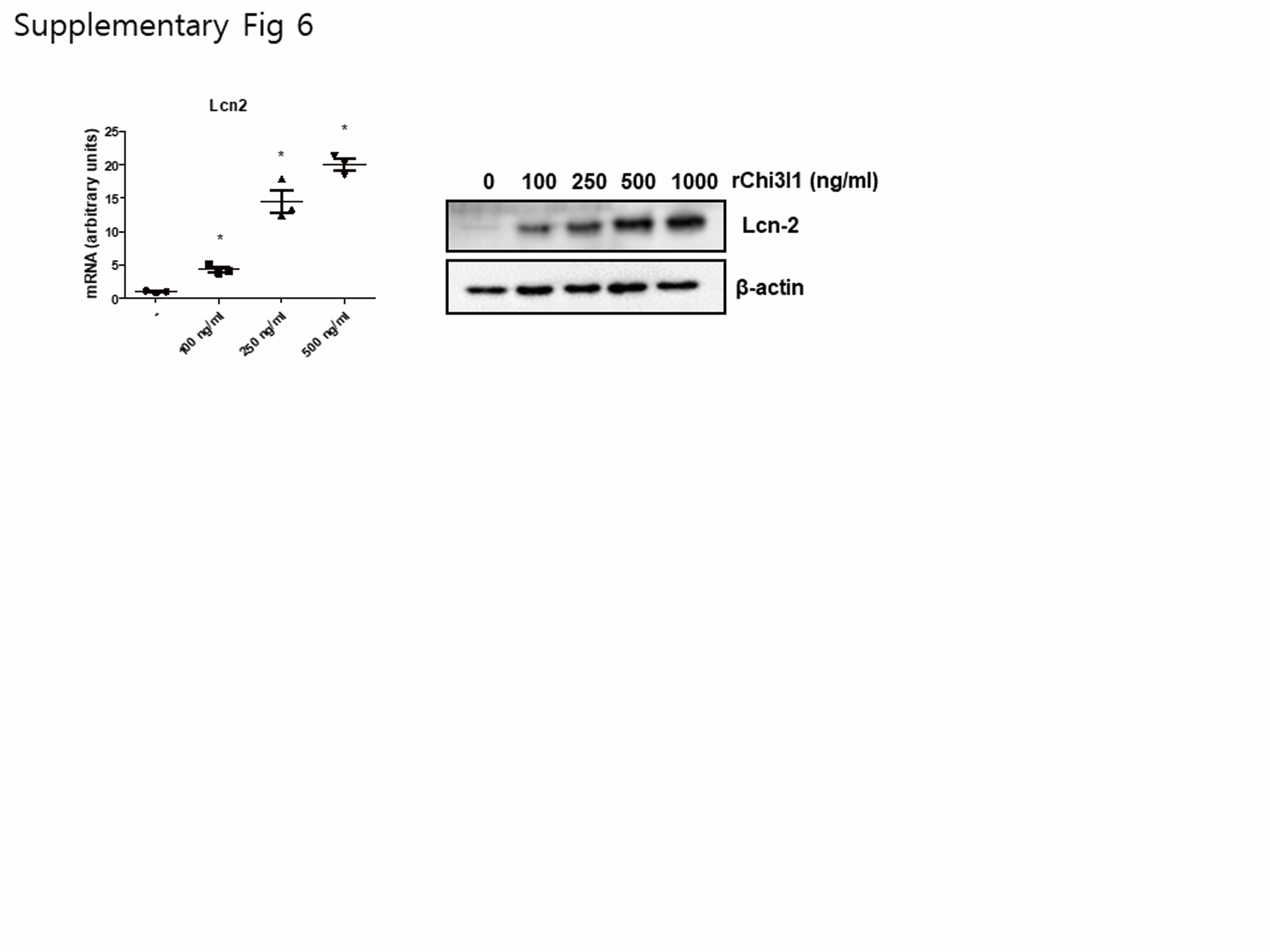

Supplement: Supplementary file 1 [file Image6.tif]

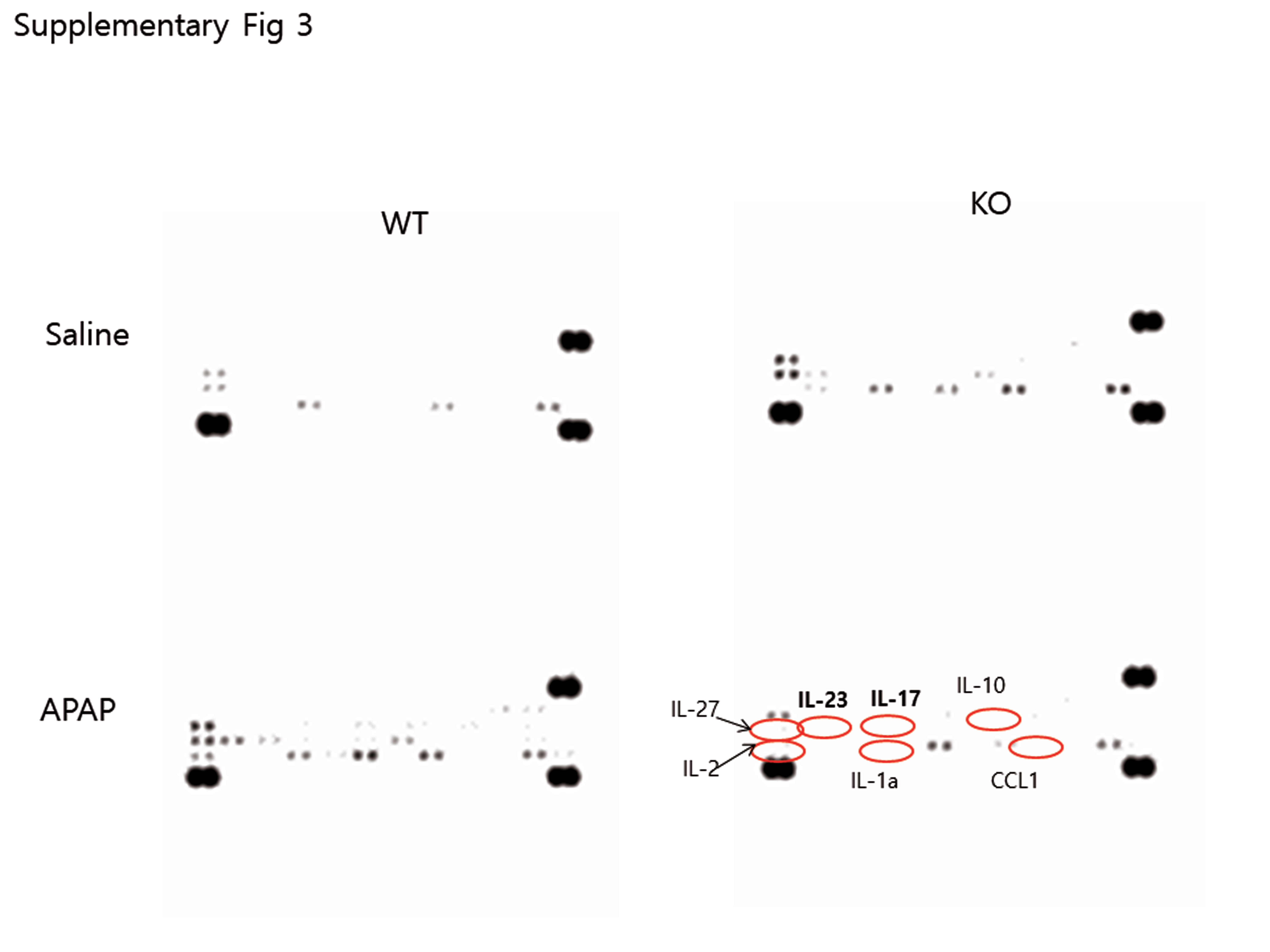

Supplement: Supplementary file 2 [file Image3.tif]

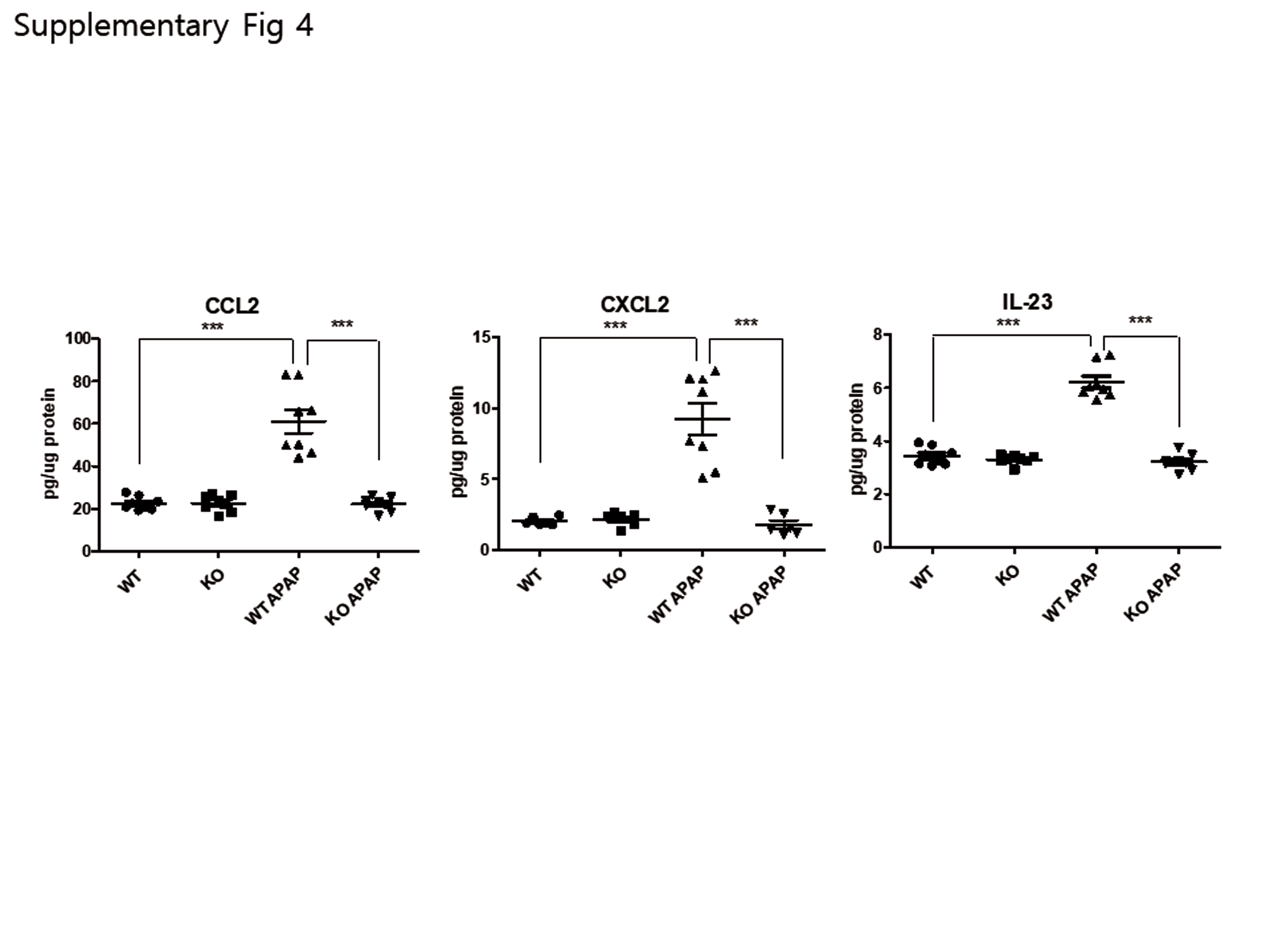

Supplement: Supplementary file 3 [file Image4.tif]

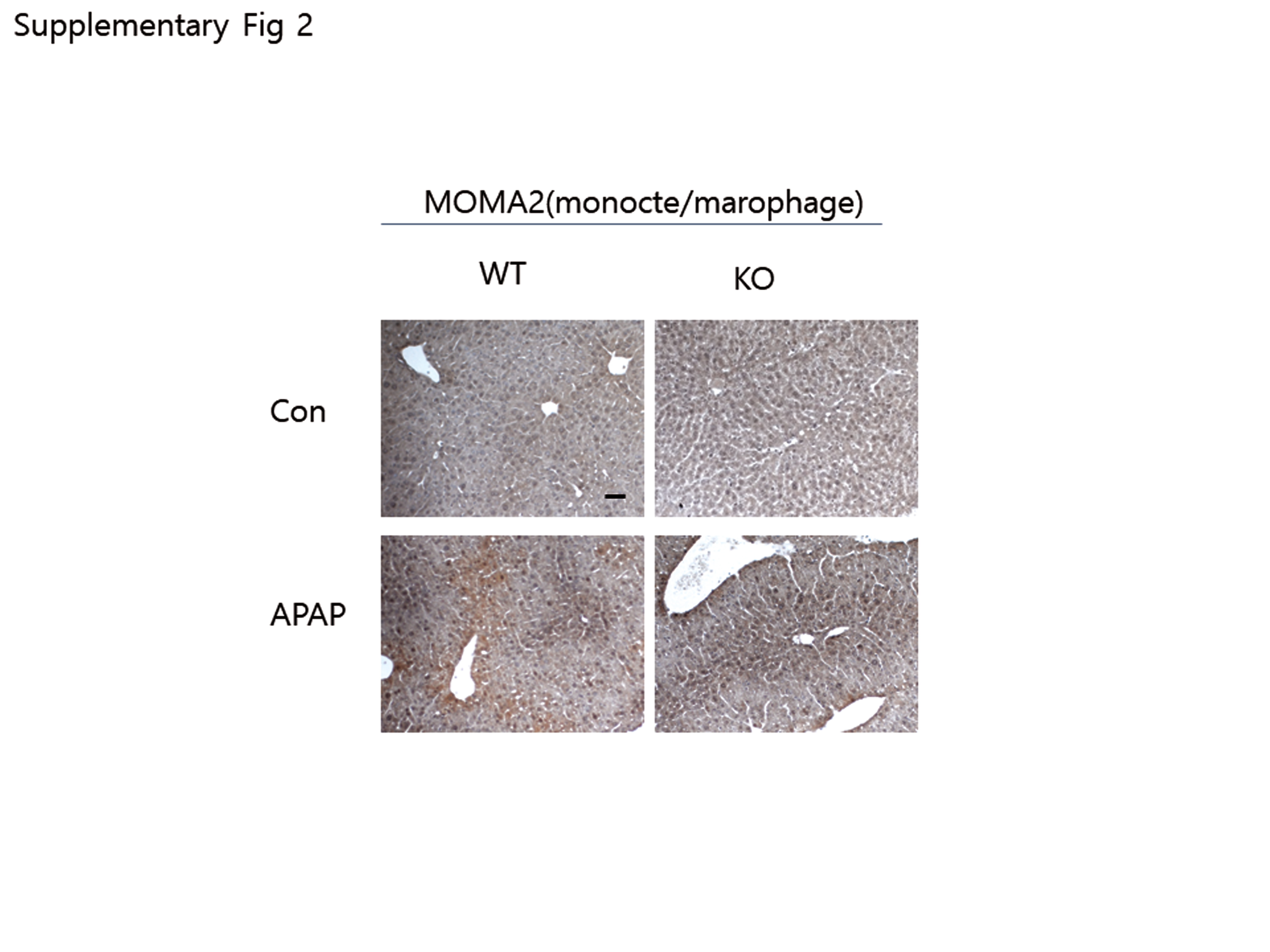

Supplement: Supplementary file 4 [file Image2.tif]

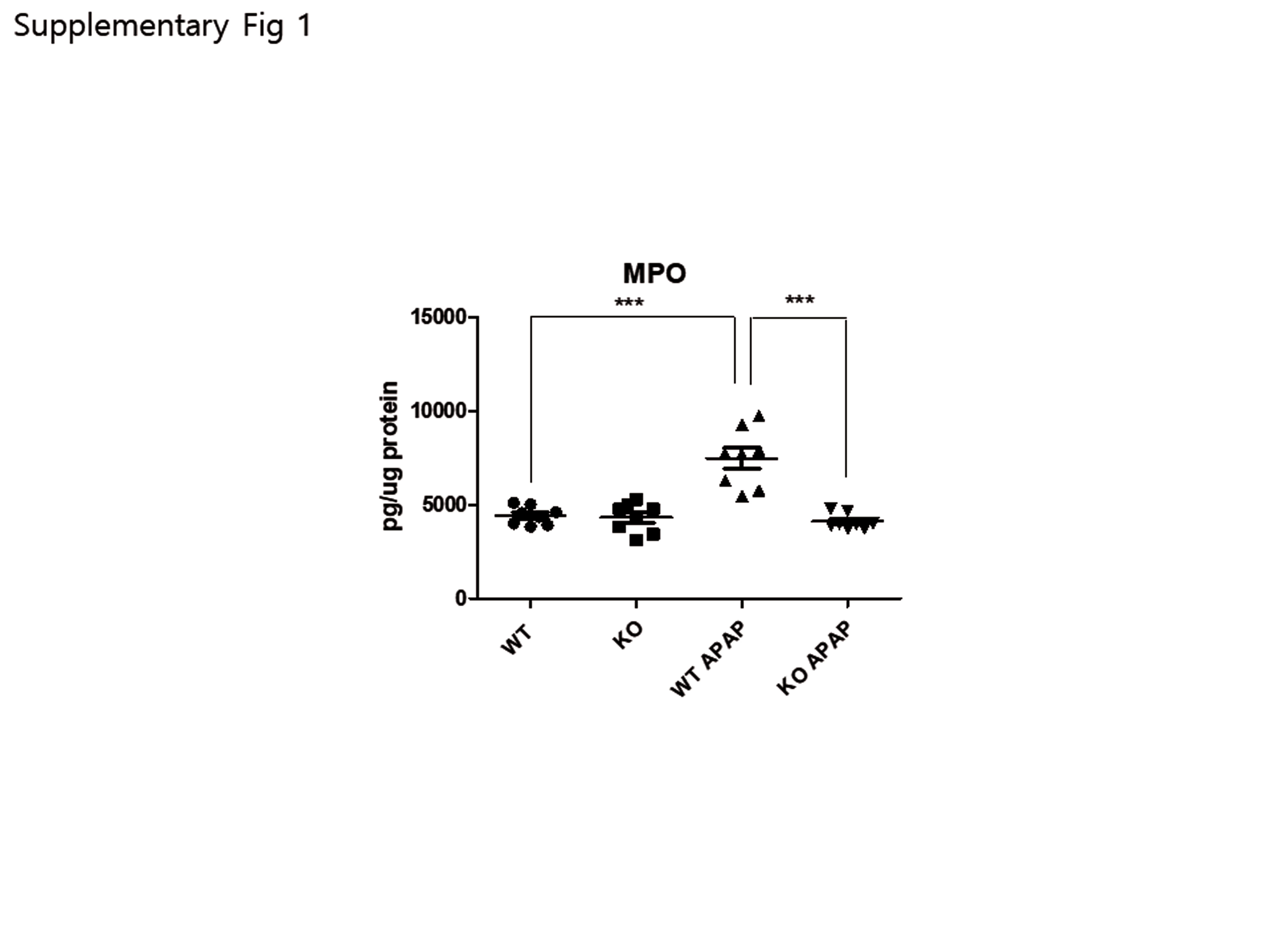

Supplement: Supplementary file 5 [file Image1.tif]

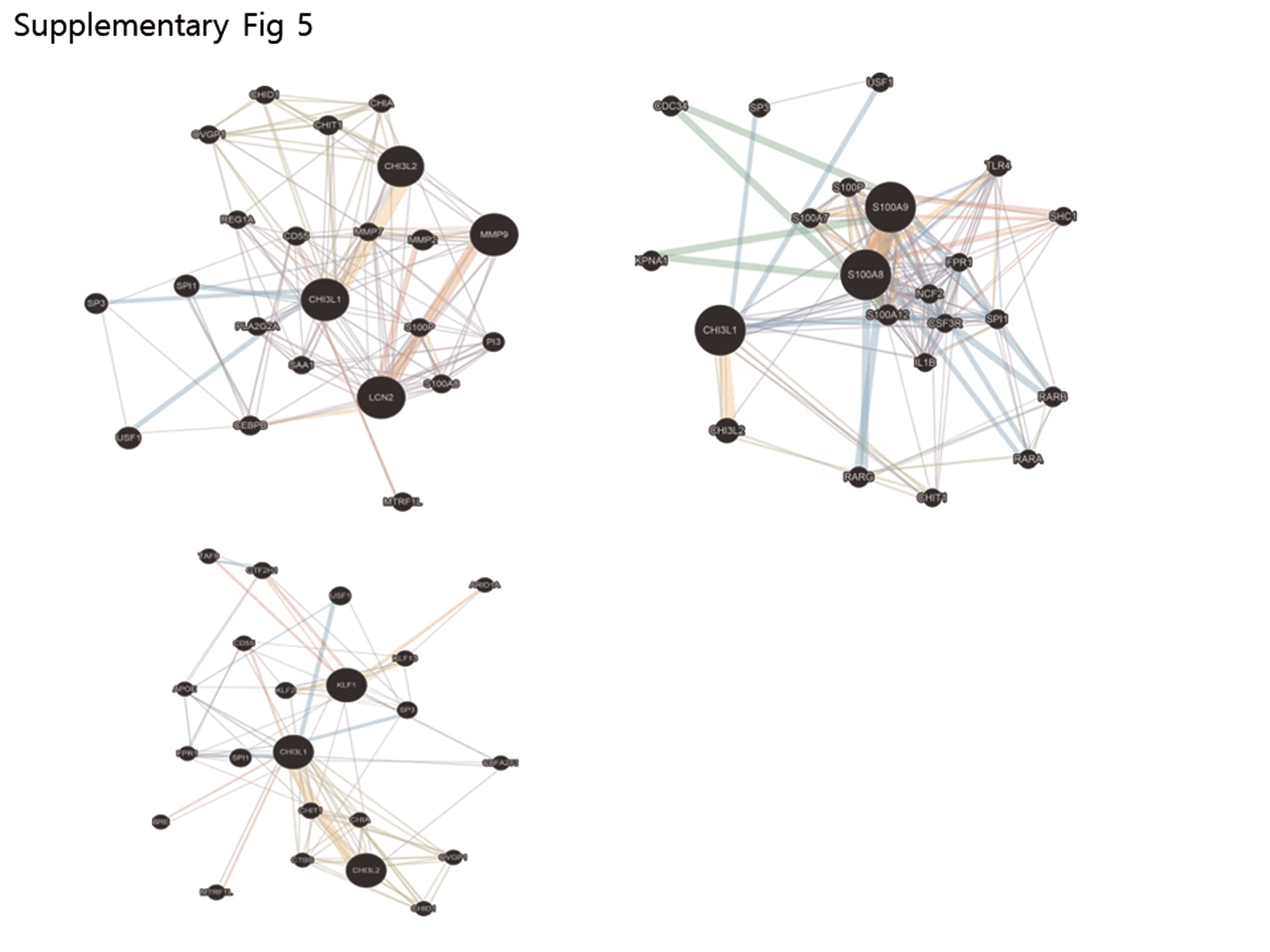

Supplement: Supplementary file 6 [file Image5.tif]
