# Supplementary material for: Chitinase 3-like protein 1 deficiency ameliorates drug-induced acute liver injury by inhibition of neutrophil recruitment through lipocalin-2
Source: Front Pharmacol. 2025 Mar 24;16:1548832. doi: 10.3389/fphar.2025.1548832 (PMC11973357; doi:10.3389/fphar.2025.1548832)
Supplement: Supplementary file 7 [file DataSheet1.docx]

**Supplementary**

Supplementary Table 1.

|  | Normal (n=19) | Patients (n=20) |
| --- | --- | --- |
| Age (years, mean±sd) | 24.75±1.222 | 56.30±3.484 |
| Total protein (g/dl, mean±sd) | 6.953±0.07737 | 6.370±0.1292 |
| ALT (U/L, mean±sd) | 29.26±4.795 | 628.0±113.7 |
| AST (U/L, mean±sd) | 23.79±3.055 | 646.2±175.4 |
| ALP (U/L, mean±sd) | 72.00±2.601 | 125.1±13.30 |
| γGT (U/L, mean±sd) | 26.15±2.708 | 214.8±50.29 |
| Bilirubun (mg/dl, mean±sd) | 0.7700±0.07633 | 3.860±1.134 |

ALP, alkaline phosphatase; ALT, alanine aminotransferase; AST, aspartate transferase; γGT, γ-glutamyltransferase

**Supplementary Table. 1. Clinical characteristics of Normal and hepatotoxicity patients in the study.**

**
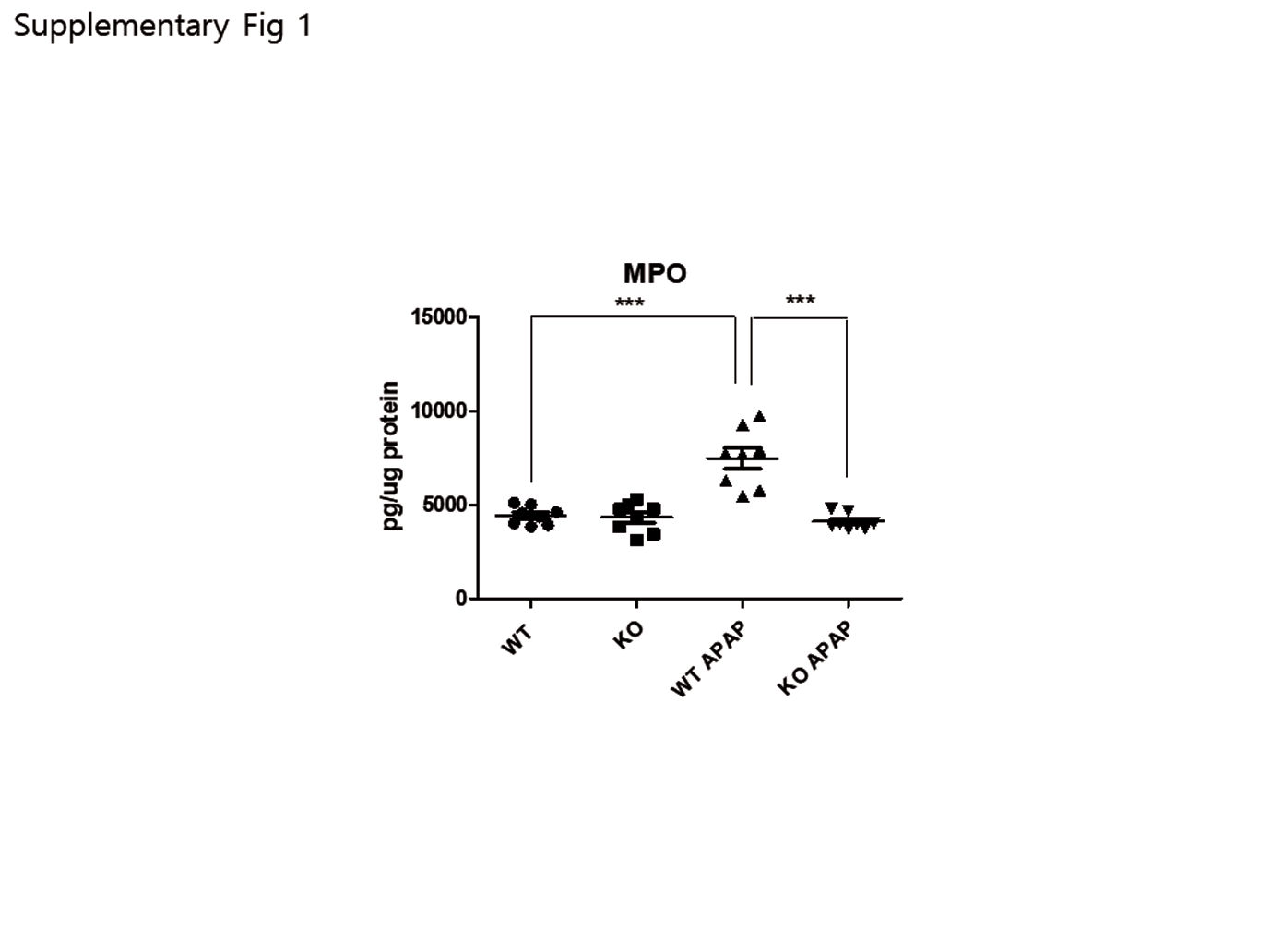
**

**Supplementary Fig. 1. The protein expression of MPO in the livers of WT and KO mice with or without APAP administration (500mg/kg). n=8 per group; means ± SEM, ^***^*P* < 0.001.**

**
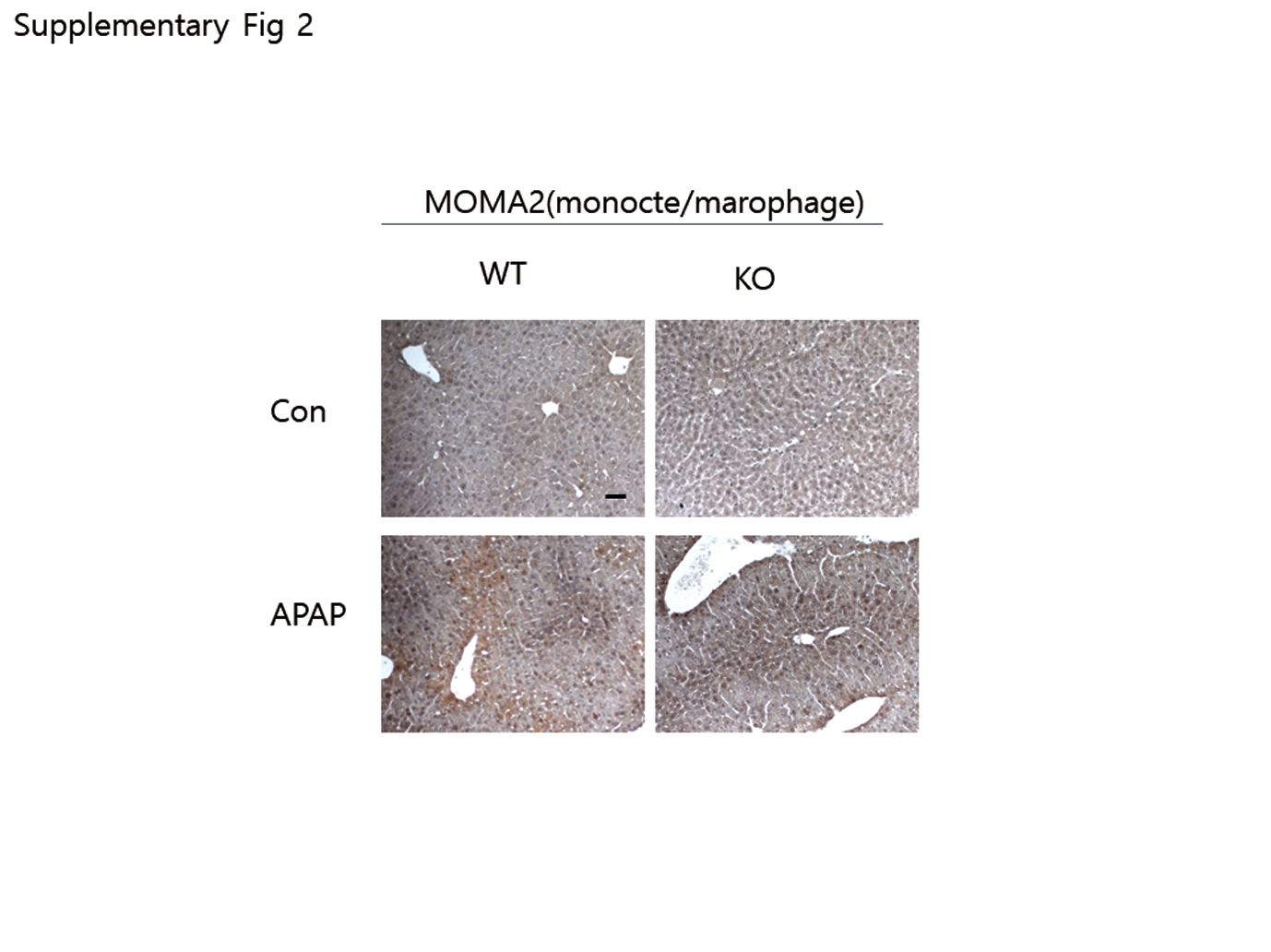
**

**Supplementary Fig. 2. Immunohistochemistry of monocytes/macrophages (MOMA2) in the liver of WT and KO mice with or without APAP administration (500 mg/kg) (Scale bars, 100μm).**

**
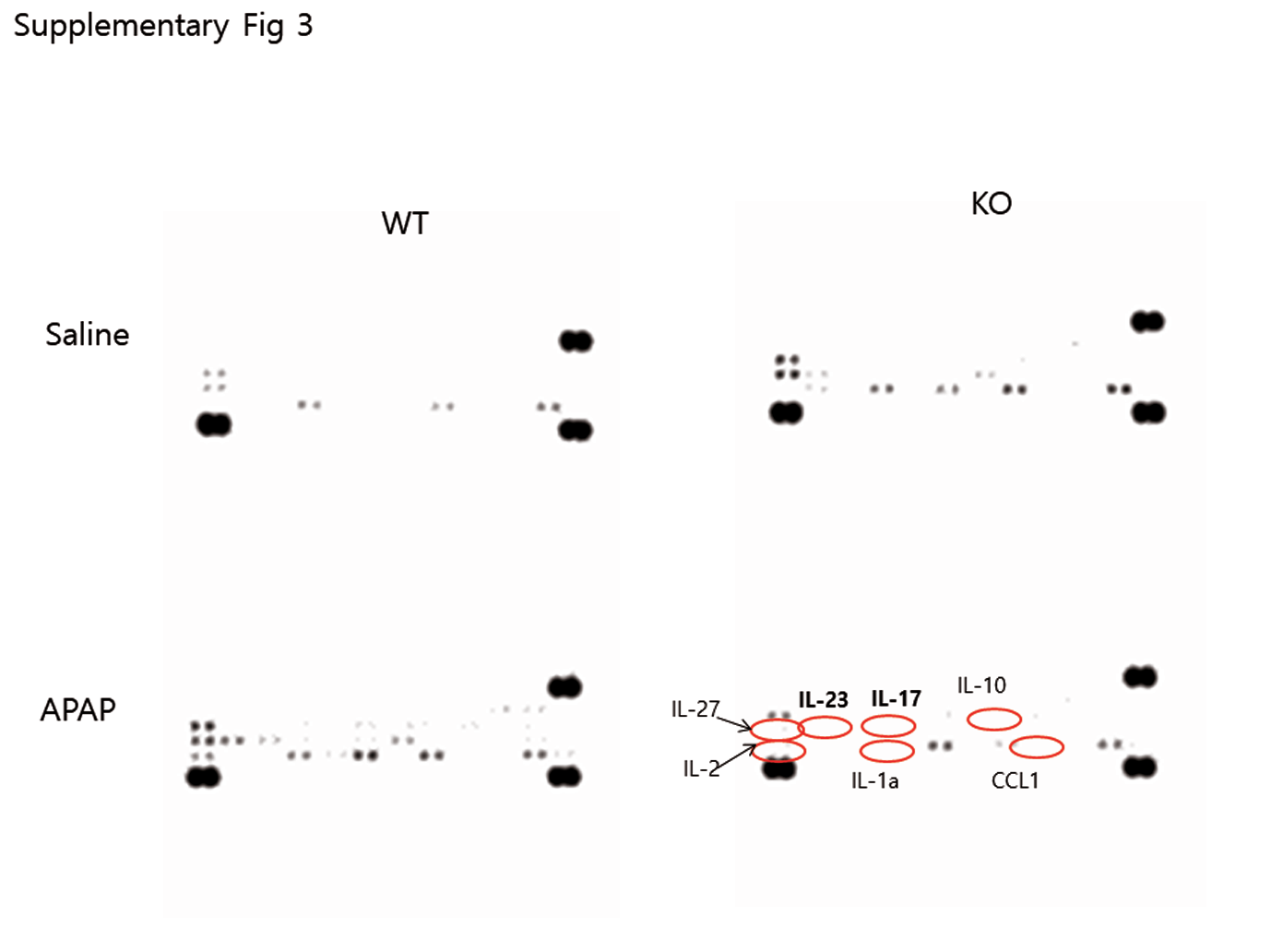
**

**Supplementary Fig. 3. Cytokine/chemokine array in the liver of WT and KO mice with or without APAP administration (500 mg/kg).**

**
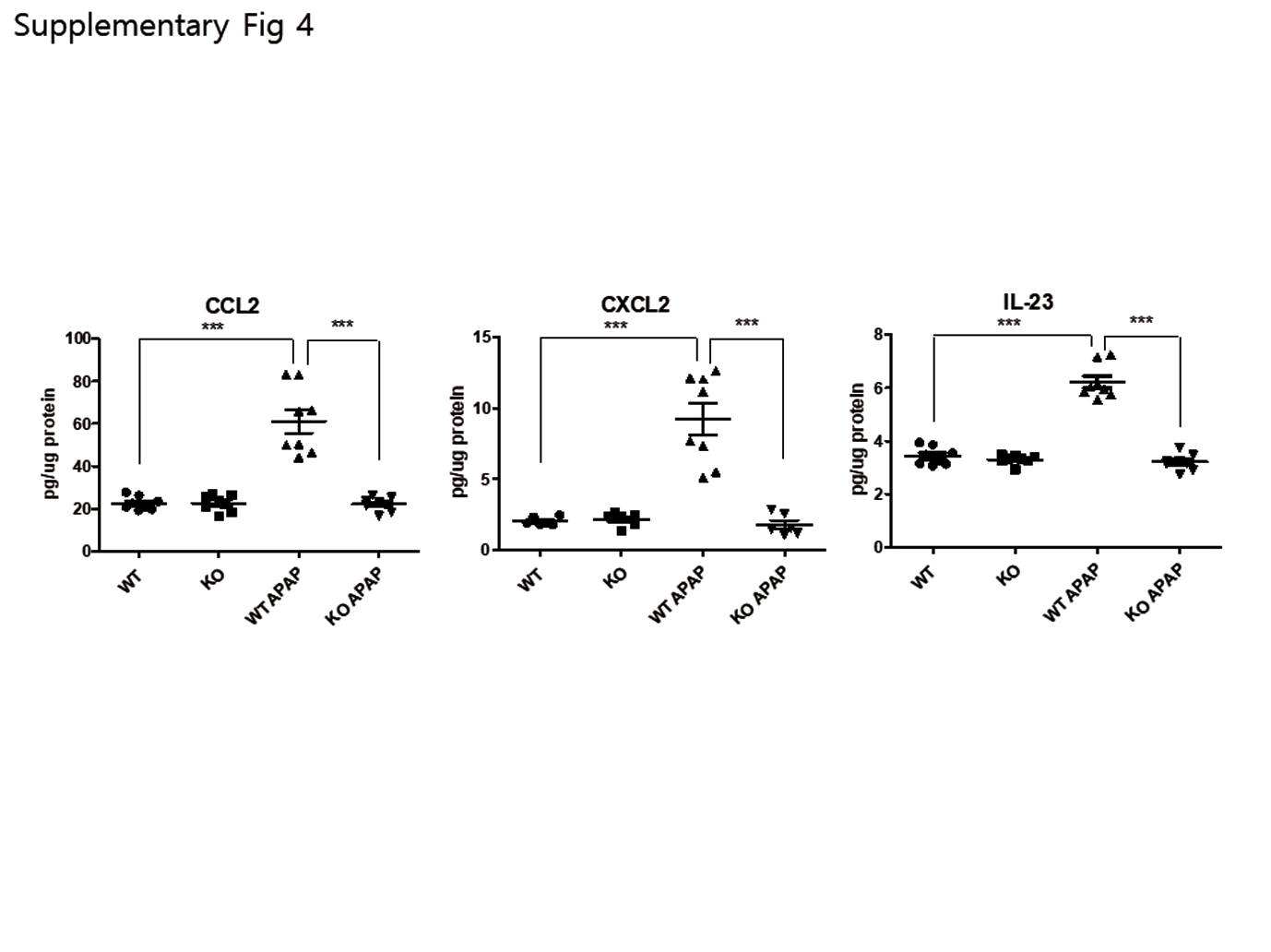
**

**Supplementary Fig. 4. The protein expression of Ccl2, Cxcl2, Il-23 of the liver of WT and KO with or without APAP administration (500mg/kg). n=8 per group; means ± SEM, ^***^*P* < 0.001.**

**
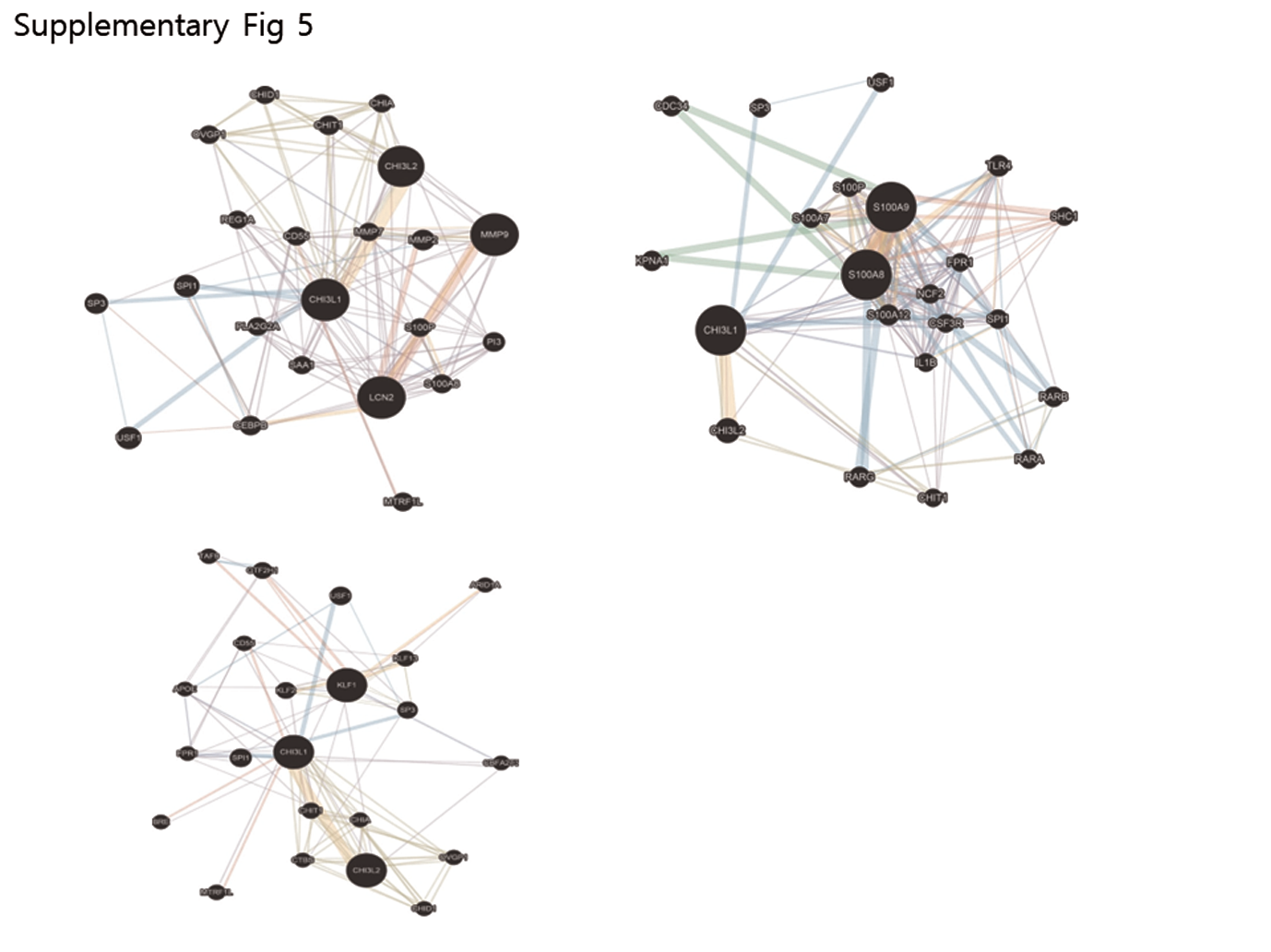
**

**Supplementary Fig. 5. Correlation between Chi3L1 with Lcn2, S100A8, and Klf1 using GENEMANIA program.**

**
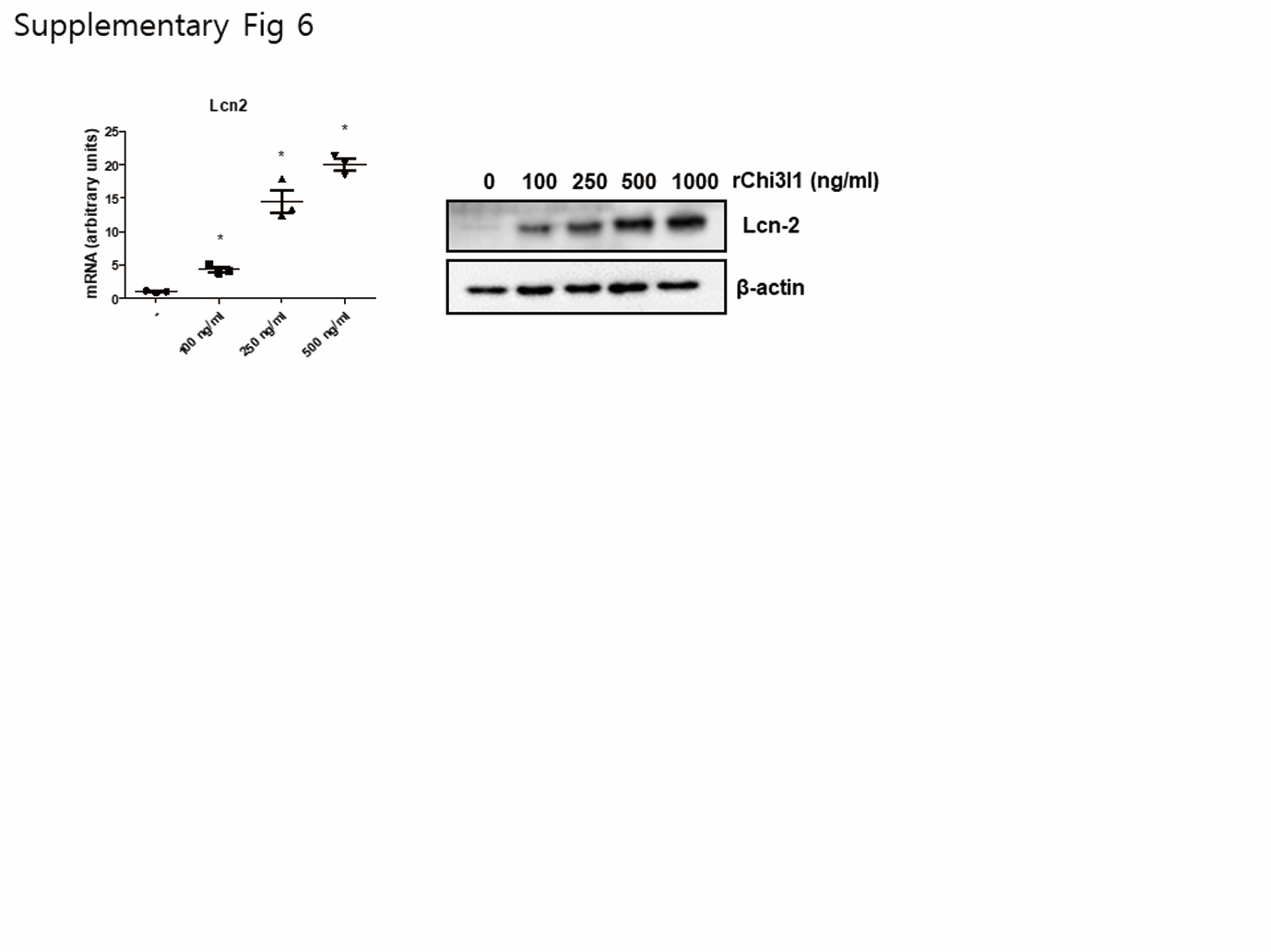
**

**Supplementary Fig. 6. The mRNA expression and immunoblot of Lcn2 in Kupffer cells dose-dependently treated with rChi3l1.**
